# Models of Cultural Reproduction as Drivers of Socio-cultural Resilience: Insights from a Systematic Review

**DOI:** 10.12688/f1000research.174513.1

**Published:** 2026-01-16

**Authors:** Mifdal Zusron Alfaqi, Moh Fadli, Alfi Haris Wanto, Tjahjanulin Domai, Akil Fitra Sholakodin

**Affiliations:** 1 Doctoral Program in National Resilience Science, Universitas Brawijaya, Malang, East Java, 65145, Indonesia; 2 Constitutional Law, Universitas Brawijaya, Malang, East Java, 65154, Indonesia; 3 Public Administration Science, Universitas Brawijaya Fakultas Ilmu Administrasi, Malang, East Java, 65145, Indonesia; 4 Sociology, Universitas Indonesia Fakultas Ilmu Sosial dan Ilmu Politik, Depok, West Java, 16424, Indonesia

**Keywords:** cultural reproduction, socio-cultural resilience, cultural capital, digitalization, social systems.

## Abstract

**Aim:**

To examine the determinants influencing cultural reproduction, comprehend the fundamental concepts of cultural reproduction, and evaluate the model of cultural reproduction as a manifestation of socio-cultural resilience.

**Materials and Methods:**

The research employed the Systematic Literature Review (SLR) methodology, utilizing the PRISMA protocol and the PICO framework. Data were sourced from Scopus, Web of Science, ProQuest, and Google Scholar, chosen based on inclusion criteria (2015–2025, empirical articles, pertinent to socio-cultural resilience). Out of the 688 initial articles, 143 studies satisfied the criteria. The Mixed Methods Appraisal Tool (MMAT) was used to assess quality, and NVivo 14 was used for thematic synthesis to find patterns of direct relationships, mediation, and moderation between factors.

**Results and discussions:**

The analysis identified eight principal factors: education, politics, family, economy, media, technology, religion, and community. Politics (15.3%) and education (17.6%) were the most important factors. Cultural reproduction operates through micro–macro interactions involving direct relationships (family, religion, community), mediation (education, media, politics, technology), and moderation (economics, technology). The resulting integrative model elucidates the way this amalgamation of factors enhances socio-cultural resilience via conservation and value adaptation.

**Conclusions:**

Cultural reproduction serves both as a means of value preservation and as an adaptive mechanism that enhances social resilience. This research enhances Bourdieu’s theory by incorporating digital and participatory dimensions, providing a conceptual foundation for sustainable cultural policy.

## Introduction

The phenomenon of cultural reproduction is garnering heightened global attention due to the escalating threat to cultural diversity. Due to globalization, urbanization, and socio-political pressures, almost half of the world’s 7,000 languages are in danger of dying out. ( Bromham et al., 2022) emphasize that threats to languages are on the rise, with a forecasted swift reduction in linguistic diversity across various continents. Digitalization has also changed how people participate in culture. ( Janssen et al., 2024) did a survey in nine European countries and found that cultural engagement is now mostly about having access to digital information, audiovisual entertainment, and making and sharing content online. This change, along with the problems of climate change and conflict, makes it even more important to look at cultural reproduction as a way to build socio-cultural resilience.

The urgency of this issue stems from its significant social ramifications. The decline of cultural reproductive mechanisms—like less passing down of traditions from one generation to the next, commodification without giving communities more power, and language loss—puts communities’ ability to adapt and stay together at risk. More and more people are recognizing the importance of intangible cultural heritage (ICH) in meeting this challenge. ( McDermott & Craith, 2024) contends that intangible cultural heritage (ICH) facilitates climate change adaptation by incorporating cultural practices into resilience strategies. Concurrently, ( Sun et al., 2024) demonstrate that sustainable livelihood frameworks can effectively integrate ICH into community resilience planning. Additionally, ( Seduikyte et al., 2025) underscore the significance of participatory governance in enhancing resilience by connecting cultural heritage policies with community involvement. Without a focused model of cultural reproduction that encompasses intergenerational transmission, digital engagement, and institutional backing, socio-cultural vulnerabilities are anticipated to escalate.

Several empirical studies have endeavoured to address this question. ( Ghahramani et al., 2020) investigated Gullah Geechee communities in the United States and discovered that cultural heritage functions as a source of resilience when associated with community-based tourism and participatory governance. ( Lv et al., 2025) examined panel data from China (2013–2022) and demonstrated that synergistic interactions between intangible cultural heritage (ICH) and tourism can facilitate sustainable development through dynamic reciprocal relationships. Janssen et al. (2024) also say that digital cultural participation changes the way culture is passed down. Nonetheless, a considerable research deficiency exists: the majority of studies remain localized, sector-specific, or concentrated on a singular variable. Integration across domains, including intergenerational transmission, digital practices, and policy ecosystems, is still insufficient. This gap highlights the necessity for a holistic model that elucidates causal relationships and mechanisms of cultural reproduction as the foundation of socio-cultural resilience.

This study seeks to examine the determinants influencing cultural reproduction, elucidate its fundamental concepts, and formulate a model of cultural reproduction as a manifestation of socio-cultural resilience. This study holds significant theoretical and practical implications. This research addresses a gap in the literature by providing an integrative framework that links cultural practices, digital infrastructure, and legacy policy. This research offers practical strategic insights for policy formulation, particularly in the areas of intangible cultural heritage (ICH) conservation, participatory governance design, and the development of resilience indicators. This research offers an adaptive and integrative model of cultural reproduction, thereby aiding in the preservation of cultural values and enhancing the socio-cultural resilience of communities amid global transformations.

## Methods

The methodology of this study employs the Systematic Literature Review (SLR) approach, selected for its capacity to deliver a thorough synthesis of diverse empirical studies concerning cultural reproduction and socio-cultural resilience. SLR enables researchers to identify, assess, and synthesize pertinent evidence in a clear and reproducible manner ( Snyder et al., 2024). Consequently, this methodology is suitable for addressing the research deficiencies identified in the introduction and for providing a comprehensive understanding across various domains. This study employs the Population, Intervention, Comparison, Outcome (PICO) framework to elucidate the SLR framework and to formulate research questions. The following Table 1 shows the details:

**Table 1.** PICO analysis framework.

Component	Description
Population	Communities and social groups involved in cultural reproduction practices, both local and transnational
Intervention	Mechanisms, strategies, or policies that affect cultural reproduction (intergenerational transmission, digital participation, cultural heritage policies)
Comparison	Differences in geographical, temporal, or sectoral contexts in the application of cultural reproduction
Outcome	Socio-cultural resilience, social cohesion, and adaptive capacity of communities

The literature search strategy is systematically designed using several major academic databases, namely Scopus, Web of Science, ProQuest, and Google Scholar. The search process is carried out with a combination of keywords and Boolean operators as follows:

(“cultural resilience” OR “cultural sustainability” OR “cultural adaptation” OR “cultural preservation”) AND (“community” OR “society” OR “group” OR “population”) AND (“identity” OR “heritage” OR “tradition” OR “values”) AND (“adaptation” OR “response” OR “recovery” OR “transformation”) AND (“disaster” OR “crisis” OR “change” OR “challenge”)

The search results are then exported for further filtering.

Inclusion and exclusion criteria are established to ensure the relevance and quality of the sources used. The following Table 2 provides a breakdown of these criteria:

**Table 2.** Data selection process.

Inclusion criteria	Exclusion criteria
Empirical articles in English	Articles in the form of reviews or non-empirical
Publications in the last 10 years (2015–2025)	Publications under 2015
Focus on cultural reproduction and socio-cultural resilience	Articles that only discuss cultural aspects with no relevance to resilience
Full access (open access or institutional access)	Articles with limited access without full availability

The PRISMA (Preferred Reporting Items for Systematic Reviews and Meta-Analyses) protocol is used to pick the articles so that each step is clear and can be traced ( Page et al., 2021). PRISMA is utilized throughout the identification phase, title and abstract screening, comprehensive text assessment, and the ultimate inclusion of the article. This protocol allows for the replication and methodological evaluation of the research flow. The latest version of the Mixed Methods Appraisal Tool (MMAT) is used to check the quality of articles. This tool lets you compare different methods, including quantitative, qualitative, and mixed methods ( Hong et al., 2018). This evaluation emphasizes the lucidity of the research design, the validity of the instrument, the pertinence of the results, and their alignment with the research objectives. The following Figure 1 provides a breakdown of these PRISMA criteria:

**Figure 1.**
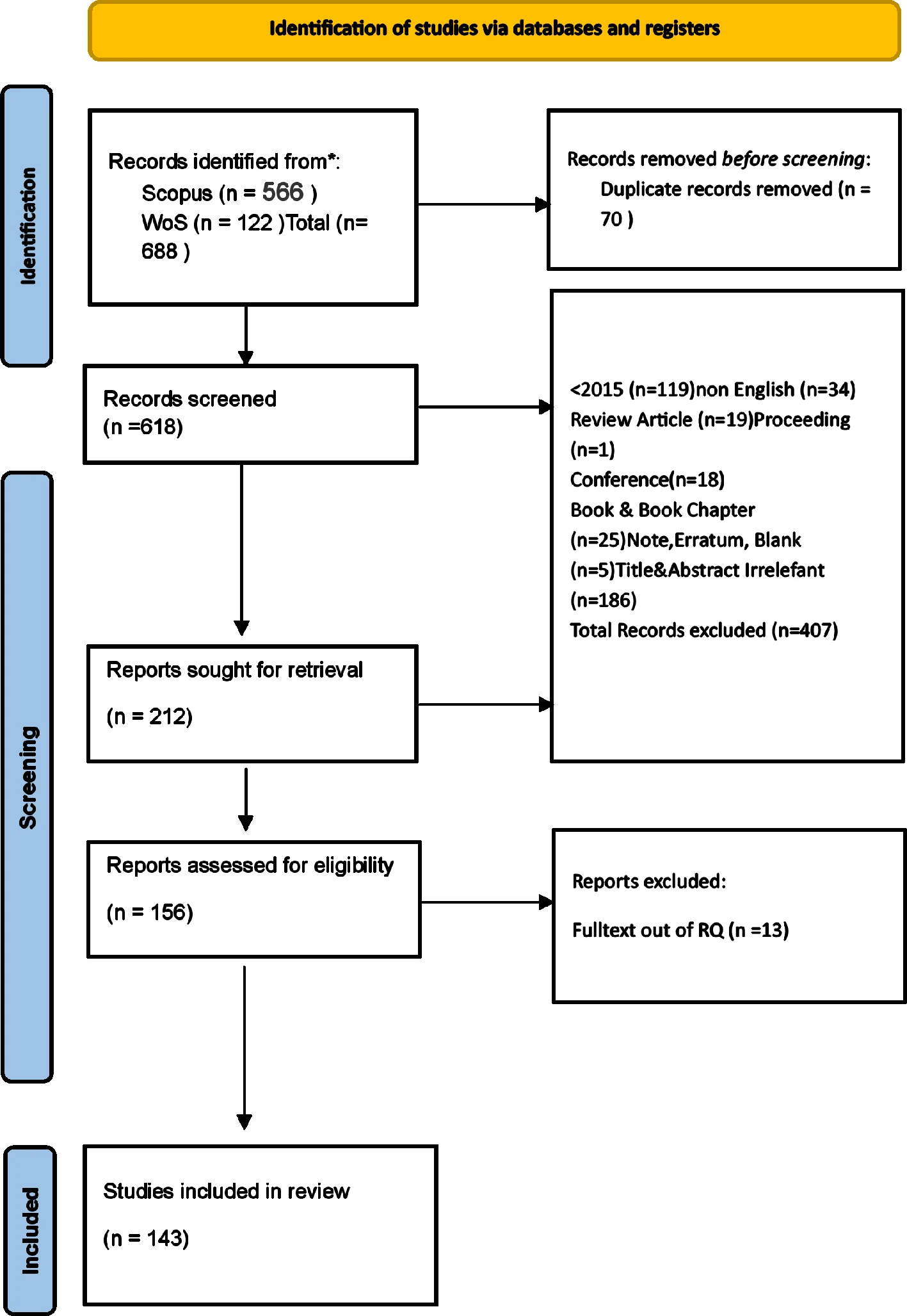
SLR’s PRISMA flows chart visualizing data selection process.

This study’s PRISMA flow diagram shows how articles were chosen in a systematic way that met the study’s criteria. We got a total of 688 articles from the Scopus (566) and WoS (122) databases. However, 70 of them were deleted because they were duplicates, leaving us with 618 articles to choose from. 407 articles were left out of the screening stage because they didn’t meet the criteria, such as being published before 2015, in a language other than English, in the form of review articles, proceedings, conferences, books, notes, or abstract titles that weren’t relevant. After that, 212 reports were looked for to download, but 56 of them could not be found, leaving 156 articles that were fully checked for eligibility. Out of these, 13 articles were removed because the full-text content did not align with the research question. So, 143 studies were finally deemed possible and added to the review. The arrow to the right in the diagram shows the articles that were published at each step of the selection process. This makes it easy to see how the process of identification, screening, and inclusion worked in this systematic review.

Data extraction entails the collection of essential information from each article, encompassing the research objectives, methodology, context, key findings, and implications. After that, NVivo 14 software was used to analyse the data using thematic analysis methods. The process involves initial coding, categorization, identification of principal themes, and thematic synthesis to address research inquiries. This method enables a comprehensive examination of the interrelationships among cultural reproductive factors and their contribution to socio-cultural resilience.

To enhance accuracy and mitigate bias, the study was executed by three researchers with a distinct allocation of responsibilities. The first researcher (P1) is in charge of the first search design and filtering of the article. The second researcher (P2) is in charge of judging the quality of the articles and getting the data out of them. The third researcher (P3) concentrated on data analysis using NVivo and the synthesis of findings. All researchers participated in triangulation discussions to guarantee the consistency of interpretation and the validity of findings. This methodology is anticipated to yield a substantial contribution to addressing research inquiries concerning concepts, factors, interrelationships among factors, and cultural reproduction models as expressions of socio-cultural resilience. A methodical, open, and evidence-based approach lays the groundwork for generating findings that are both academically and practically sound.

## Results

Several studies emphasize the important role of cultural reproduction in increasing socio-cultural resilience ( Ghahramani et al., 2020; Lv et al., 2025; Gramsci, 1971; Bourdieu, 1986; Castells, 2000). This study identifies the main factors that influence cultural reproduction, such as education, politics, family, and technology, as well as how these factors contribute to the adaptation of cultural values in response to dynamic social contexts. In particular, education was identified as the most dominant factor, responsible for 17.6% of cultural reproduction, which strengthens social structures through curriculum and pedagogy ( Bourdieu & Passeron, 1990). Likewise, politics and the family have a significant impact, with politics controlling cultural discourse through state policies and ideologies ( Gramsci, 1971), while the family serves as the main agent in transmitting cultural norms and values between generations ( Bourdieu, 1977). In addition, digital technologies are increasingly seen as mediators that accelerate cultural transmission, creating new channels for cultural engagement and adaptation through global networks ( Castells, 2000). These findings confirm that cultural reproduction is not a static process, but rather one that develops through the interaction of micro and macro factors, with each factor playing a unique role in strengthening social resilience. The following Figure 2 explains the percentage of articles that are analyzed in the context of cultural reproduction.

**Figure 2.**
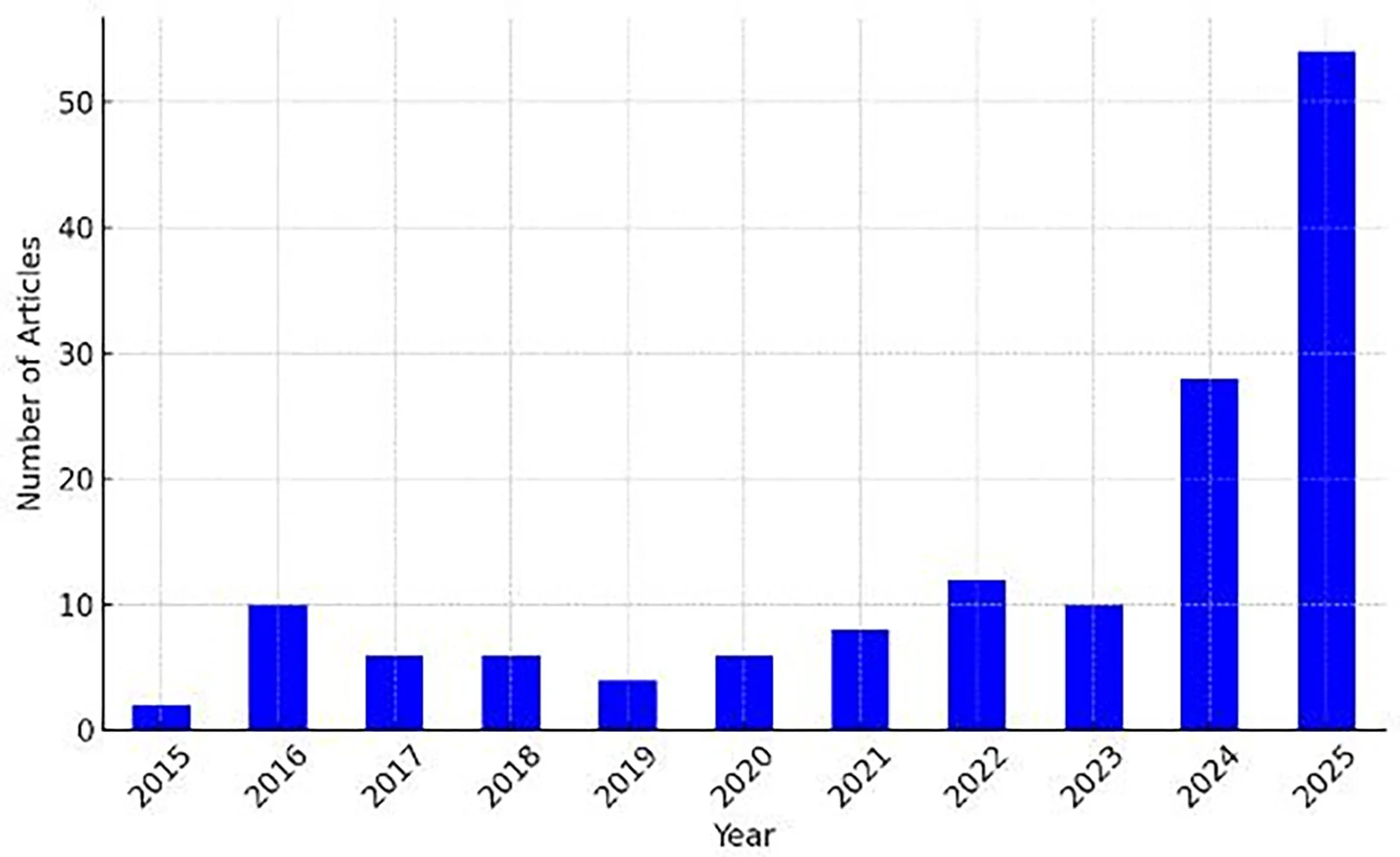
Percentage of articles analyzed.

The article distribution bar graph for each year shows that the number of publications has been going up since 2015. The number of articles varies which shows how academic interest in the research topic being studied has changed over time. The number of articles was still low in the first few years after 2015. After that, the graph showed a steady rise, which meant that research was getting more intense and issues in the academic world were becoming more important. Researchers may pay more attention to certain years when there are peaks in certain areas. These peaks may be due to changes in theory, policy, or global events. In the meantime, small changes from year-to-year show how the focus of research has changed and how the number of publications in the database has changed. Overall, this graph shows a positive trend, with more recent publications on the rise. This shows that research topics are still an important part of modern scientific discourse. The following Figure 3 shows the novelty of the studies that have been analyzed in the VosViewer application.

**Figure 3.**
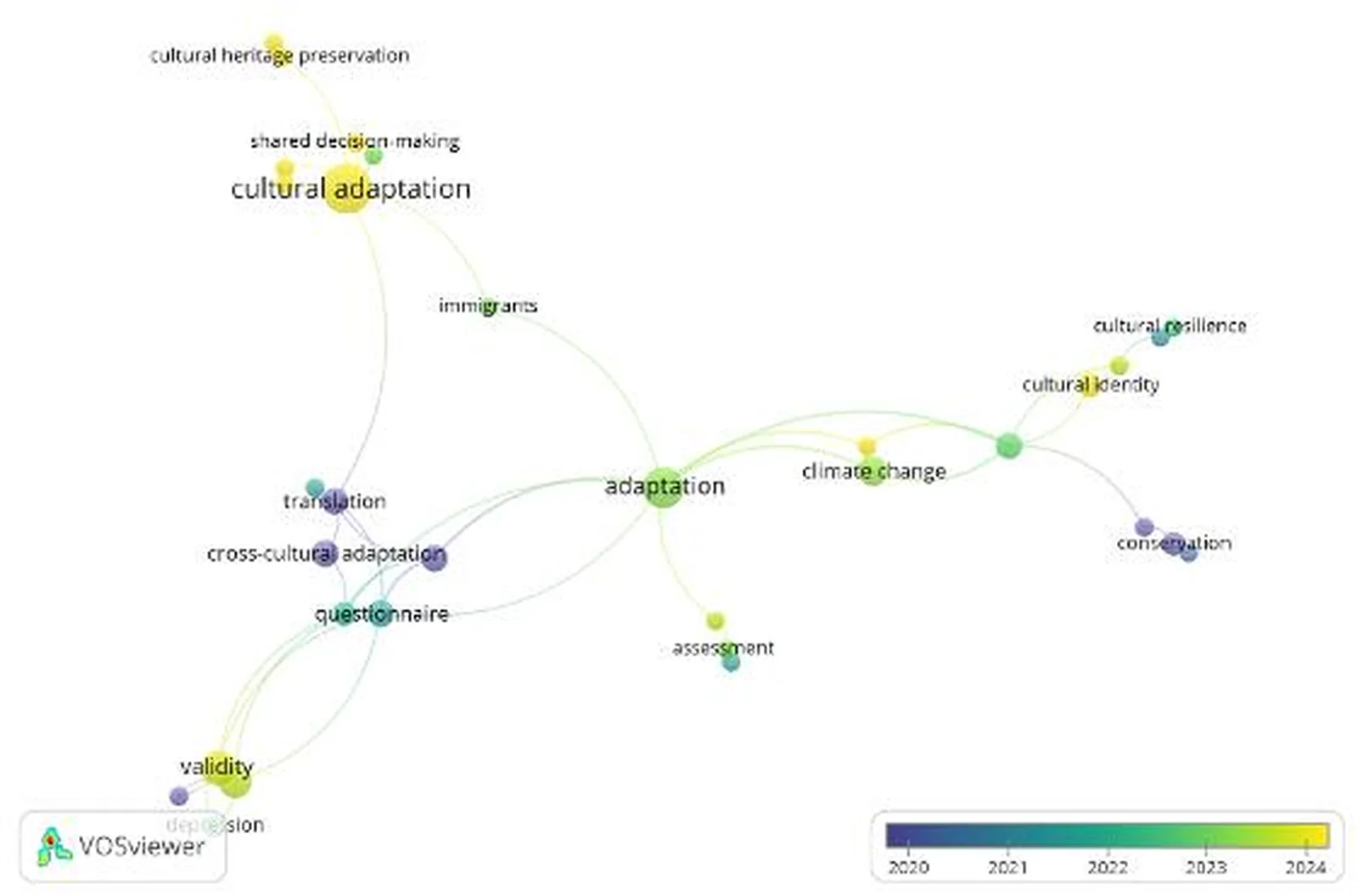
Changing trends in research topics on Cultural Reproduction.

This VOSviewer overlay visualization shows how research topics about adaptation have changed over time, with colors that change based on the year of publication (purple = 2020, blue = 2021, green = 2022, yellow = 2023–2024). The central term “adaptation” serves as the principal nexus, extending to diverse domains, including cultural adaptation, climate change, assessment, and cultural identity. The temporal pattern shows that the first research (2020–2021, purple-blue) was more about methodological issues like translation, questionnaires, and validity. As we move into the 2022 (green) period, people are more interested in social and environmental issues like climate change, immigrants, and conservation. The most recent trend (2023–2024, in yellow) focuses on the cultural aspect, especially cultural adaptation, cultural resilience, cultural heritage preservation, and shared decision-making. This shows that research is moving away from a technical approach and toward a multidisciplinary understanding of identity, sustainability, and community participation. This pattern illustrates the progression of the theme from a conceptual framework to the implementation of adaptation within a more expansive cultural and environmental context.

### Cultural reproductive factors

Factors that influence cultural reproduction span a wide range of dimensions, ranging from education, politics, economics, family, to media, religion, technology, and social community. Each factor plays a role in maintaining, transmitting, and transforming cultural values, norms, and identities across generations. Education and politics occupy a dominant position due to their role in shaping social structure and legitimacy, while other factors serve as complementary supporting arenas. A comprehensive picture of the distribution of these factors can be seen as described in Table 3.

**Table 3.** Distribution of keywords based on cultural reproduction factors.

Factor	Description	Verbatim quotes	Number of relevant citations	List of citations
Family	A primary socialization agent who instills cultural values, norms, languages, and practices from an early age.	“The family is the primary site where cultural capital is transmitted and embodied” ( Bourdieu, 1977, p. 87).	12	( Alhajj et al., 2017; Chrzan-Rodak et al., 2024; Deffner & McElreath, 2022; Sémah, 2017)
Education	Schools reproduce the habitus and legitimacy of the dominant culture through pedagogical curricula and practices.	“Education systems contribute to the reproduction of the structure of power relations and symbolic relations between classes” ( Bourdieu & Passeron, 1990, p. 54).	15	( Alhajj et al., 2017; Biben et al., 2025; Lui & Johnston, 2019; Pardoel et al., 2022)
Mass Media	The media disseminates representations, symbols, and ideologies that defend or challenge hegemony.	“Media culture provides the materials for constructing views of the world, behavior, and identities” ( Kellner, 1995, p. 1).	10	( Binion et al., 2024; Coffey & Noble, 1996; W. Liu, 2017; Qamar & Ibrahim, 2024; Sultan et al., 2024)
Religion	Religion nurtures rituals, morality, and collective identities that are passed down through generations.	“Religion is a system of symbols which acts to establish powerful, pervasive, and long-lasting moods and motivations” ( Geertz, 1973, p. 90).	8	( Geertz, 1981) ( Anwar et al., 2025; Kline et al., 2018; Ragab et al., 2021; Seabra et al., 2025; Zhu & Feng, 2024)
Economics	Economic structure and capital distribution affect access to cultural resources.	“The forms of capital … are at the root of the reproduction of social hierarchies” ( Bourdieu, 1986, p. 241).	11	( Casciani et al., 2022; Fanari & Kim, 2025; Gibson et al., 2025; Kudelić et al., 2025; Paplikar et al., 2022; Pascoe & McLeod, 2016)
Politics and Power	The state and political institutions influence cultural reproduction through ideology, laws, and policies.	“The supremacy of a social group manifests itself in two ways, as ‘domination’ and as ‘intellectual and moral leadership’” ( Gramsci, 1971, p. 57).	13	( Chiang & Chang, 2018; Deffner & McElreath, 2022; Huang et al., 2025; Martins & Sá, 2025; Yerlanova et al., 2025)
Technology and Digitalization	Digital platforms accelerate cultural reproduction through algorithms and global networks.	“The network society is a social structure based on networks activated by digital information and communication technologies” ( Castells, 2000, p. 21).	9	( Koyuncu et al., 2020; Nur et al., 2025; Qamar & Ibrahim, 2024; Self et al., 2022; Sultan et al., 2024)
Social Environment and Community	Interaction within the community creates a social space for cultural reproduction and transformation.	“Communities are to be distinguished, not by their falsity/genuineness, but by the style in which they are imagined” ( Anderson, 1991, p. 6).	7	( Kuzmanov, 2025; Lu & Phanlukthao, 2025; Pagán et al., 2025)

The study of donut tables and diagrams reveals the allocation of literary focus on eight primary factors that affect cultural reproduction. The educational factor comprises the largest segment, with 15 citations or 17.6% of the total, affirming that educational institutions play a pivotal role in the transmission of cultural values and norms. Education serves as a formal teaching medium and a mechanism for the reproduction of social habitus that sustains class structures and social hierarchies ( Bourdieu & Passeron, 1990). The curriculum, language of instruction, and pedagogical methods systematically reinforce the legitimacy of dominant cultures, rendering education a strategic domain for perpetuating or contesting social inequality.

Thirteen citations, or 15.3%, put political and power factors in second place. This demonstrates the significance of the state and political institutions in influencing the trajectory of cultural reproduction via policies, regulations, and ideologies. Gramsci (1971) elucidates that political hegemony is executed not solely through coercive means, but also via ideological domination that compels subordinate groups to perceive dominant values as inherently natural. So, politics controls the flow of cultural discourse by giving it a symbolic legitimacy that is part of the collective consciousness of society.

Third, the family factor had 12 citations, or 14.1%, which shows that the family is the main socialization agent that passes down language, customs, and cultural values from a young age ( Bourdieu, 1977; Lareau, 2011). Cultural capital conveyed within the family establishes a basis for individuals to obtain and amass additional forms of capital, both economic and symbolic, in subsequent stages of life. The economy comes in fourth place, with 11 citations, or 12.9%. This shows that the way economic capital is distributed has a big effect on how culture can reproduce itself. Bourdieu (1986) asserted that the allocation of economic capital is intricately linked to cultural capital, as access to education, media, and political arenas is significantly influenced by the availability of economic resources.

The mass media factor was responsible for 10 citations, or 11.8%, which shows that the media is an important way to spread cultural ideas, ideologies, and group stories. The media is not impartial; it is a battleground for meaning where prevailing forces strive to maintain hegemony, while opposing forces endeavor to contest it ( Kellner, 1995). Also, technology and digitalization are at 9 citations or 10.6%, which shows how information technology has changed the way culture is passed down from one generation to the next in the modern world ( Castells, 2000). Digital platforms, algorithms, and transnational networks facilitate the transcendence of cultural boundaries, engendering novel modes of interaction that frequently result in identity hybridization.

The religious factor had 8 citations, or 9.4%, which shows that even though society is changing quickly, religion is still the main way that moral values and a sense of community are passed down. Geertz (1973) says that religion’s symbolic system creates meanings that shape the collective consciousness through rituals, traditions, and moral teachings. Lastly, the social and community environment, with 7 citations or 8.2%, demonstrated that daily interactions within local communities, ethnic groups, and social spaces are crucial for sustaining the dynamics of cultural reproduction. The community serves as both a site for conservation and a platform for negotiating meaning and identity, which evolves in response to contextual factors ( Anderson, 1991).

The citation distribution indicates that the literature predominantly focuses on structural factors, including education, politics, and economics, that institutionally influence patterns of cultural reproduction. These factors constitute the predominant focus in academic studies, whereas community and religious factors are comparatively underemphasized. This pattern indicates that research often emphasizes macro mechanisms over macrodynamic, despite the significance of both in cultural reproduction. These results suggest that comprehending cultural reproduction necessitates a holistic approach that examines both substantial institutions and quotidian social interactions.

### Types of factor relationships with cultural reproduction

The types of relationships between factors and cultural reproduction show a diversity of mechanisms at work, ranging from direct relationships such as family, religion, and community to mediations run by education, media, politics, and technology. In addition, there is also a role of moderation played by economics and technology in strengthening or weakening the influence of other factors. This pattern shows that cultural reproduction does not take place alone, but through a combination of micro and macro mechanisms that complement each other. A comprehensive overview of this type of relationship can be seen as described in Table 4.

**Table 4.** Distribution keyword based on Factor Relationships.

Factor	Types of relationships	Description	Verbatim quotes	Citation
Family	Immediately	The family is the primary socialization agent that directly transmits values, norms, language, and habitus to the next generation.	"The family is the primary site where cultural capital is transmitted and embodied"	( Qamar & Ibrahim, 2024; Saisuk et al., 2025)
Education	Immediately	Education directly reproduces the habitus and legitimacy of the dominant culture through pedagogical curriculum and practices.	"Education systems contribute to the reproduction of the structure of power relations and symbolic relations between classes"	( Casciani et al., 2022; Grarup et al., 2019; Monroe et al., 2024; Nittas et al., 2024)
Education	Mediation	Education also acts as a mediator between the family's cultural capital and wider social opportunities.	"Schools mediate the transmission of cultural capital across generations"	( Datzmann et al., 2021; Naralieva et al., 2025; Nittas et al., 2024)
Mass Media	Mediation	The media serves as a mediator in the dissemination of cultural symbols, discourses, and representations that form the collective consciousness.	"Media culture provides the materials for constructing views of the world, behavior, and identities"	( Correa & Inamdar, n.d.; Naralieva et al., 2025; Zhao et al., 2025)
Religion	Immediately	Religion directly maintains its identity and moral values through rituals, symbols, and traditions.	"Religion is a system of symbols which acts to establish powerful, pervasive, and long-lasting moods and motivations"	( Glasbey et al., 2023; Suhodolli, n.d.; Tsfati et al., 2023)
Economics	Moderation	The distribution of economic capital does not directly create cultural reproduction, but moderates individual or group access to education, media, and other cultural resources.	"The forms of capital … are at the root of the reproduction of social hierarchies"	( Binder et al., 2025; P. Liu, 2025; Tahara et al., 2023; Yamaguchi & Mitsuhashi, 2025)
Politics and Power	Immediately	Politics shapes the reproduction of culture directly through state policies and institutions.	"The supremacy of a social group manifests itself in two ways, as 'domination' and as 'intellectual and moral leadership'"	( Binion et al., 2024; Holdar et al., 2021; Kasemets & Nugin, 2022; Lu & Phanlukthao, 2025)
Politics and Power	Mediation	Politics also mediates the legitimacy of dominant values into public consciousness through ideology.	"Ideology functions to secure the spontaneous consent of the masses"	( Biben et al., 2025; Nur et al., 2025; Self et al., 2022)
Technology and Digitalization	Mediation	Technology is a mediator in accelerating the flow of culture across borders.	"The network society is a social structure based on networks activated by digital information and communication technologies"	( Aydin et al., 2025; Gibson et al., 2025; McCarty et al., 2024; Wang & Nam, 2025)
Technology and Digitalization	Moderation	Technology also acts as a moderator that reinforces or weakens the influence of other factors.	"Digital platforms can amplify or constrain cultural flows"	( Aksoy, 2024; Paplikar et al., 2022; Zhang et al., 2025)
Social Environment and Community	Immediately	Communities directly shape cultural reproduction spaces through everyday interactions, social practices, and collective imagination.	"Communities are to be distinguished, not by their falsity/genuineness, but by the style in which they are imagined"	( Janssens et al., 2017; Kaufusi, 2024; Policastro et al., 2019; So & Ip, 2020; Tverskoi et al., 2024)

The results of the table and heatmap study show that cultural reproductive factors have different types of relationships, reflecting the diversity of mechanisms at work in the process of inheriting values, norms, and identities. First, family and religious factors have a direct relationship to cultural reproduction. The family as the primary socialization agent instills habitus, language, and norms that are automatically inherited across generations ( Bourdieu, 1986). Similarly, religion creates the reproduction of values through symbols, rituals, and belief systems that make up a collective identity ( Geertz, 1981). In this context, the family and religion are fundamental sources of the formation of cultural consciousness without additional intermediaries or mechanisms, since they are integrated in the daily practices of society.

Furthermore, the education factor occupies a dual position with a direct relationship as well as mediation. As a direct relationship, education functions to reproduce social structures through curriculum, academic evaluation, and pedagogical practices that instill dominant habitus ( Jiao et al., 2016). But education also works as a mediator by bridging cultural capital inherited from the family into a broader social system. For example, children from families with high cultural capital tend to take advantage of educational opportunities more easily to access greater social resources. Thus, education not only establishes structure, but also mediates the distribution of capital between generations.

Mass media and digital technology factors play a role mainly through mediation relationships. Mass media provides symbolic representations that shape public opinion, direct behavior, and mediate individual perceptions of social reality ( Anwar et al., 2025). Meanwhile, digital technology serves as a mediator that accelerates cultural flows across geographical boundaries, creating new forms of cultural interaction in cyberspace ( Anwar et al., 2025). These two factors confirm that cultural reproduction does not only take place in physical spaces, but also in symbolic and digital spaces that are increasingly dominant in modern society.

In addition to serving as a mediator, digital technology also acts as a moderator. Technology amplifies or weakens the effects of other factors, for example by expanding the reach of education through online platforms or changing the way mass media distributes information. In other words, technology creates conditions that regulate the intensity of the influence of other factors on cultural reproduction ( So & Ip, 2020). Similarly, economic factors also serve as moderators, as the distribution of economic capital determines the extent to which individuals or groups can access education, media, and technology. Those with greater economic resources have a greater chance of maintaining or expanding their cultural influence ( Bourdieu, 1984).

Political and power factors show a pattern of dual relationships: direct and mediated. As a direct relationship, politics directs cultural reproduction through education policies, language regulations, or national cultural policies. However, politics also functions as a mediator by channeling dominant ideologies and values into public consciousness. Gramsci (1971) emphasized that political hegemony works through a combination of coercive domination and moral-intellectual leadership, so that the state and the political elite become the main link between the power structure and the cultural consciousness of citizens.

Finally, social and community environmental factors have a direct relationship with cultural reproduction. Everyday interactions within the community create a space for the formation of a collective identity, where norms, practices, and symbols are inherited and negotiated ( Anderson, 1991). These relationships are direct because communities provide a real social arena for cultural practice, without the need for formal institutional intermediaries.

The distribution of relationships shown by the heatmap shows an interesting pattern: structural factors such as education, politics, and economics tend to play a role through mediation or moderation mechanisms, while socio-cultural factors such as family, religion, and community tend to be directly related with cultural reproduction. Media and technology occupy a transitional position, serving as mediators as well as moderators. This indicates that the process of cultural reproduction takes place through a combination of direct and indirect mechanisms, in which macro institutions play more of a role as a liaison or regulator, while microagents play a role in everyday transmission.

### Cultural reproduction

Cultural reproduction is the way that social values, norms, symbols, and practices are passed down from one generation to the next. This happens through primary agents like family, religion, and community, as well as through intermediary institutions and mechanisms like education, media, politics, economics, and technology. This process not only preserves the current social structure but also facilitates the transformation and negotiation of meaning in response to an evolving social context. There are three types of relationships between each factor: direct, mediated, and moderate. Together, these make up a complicated system for keeping culture alive. The interaction pattern of these factors is illustrated in Figure 4.

**Figure 4.**
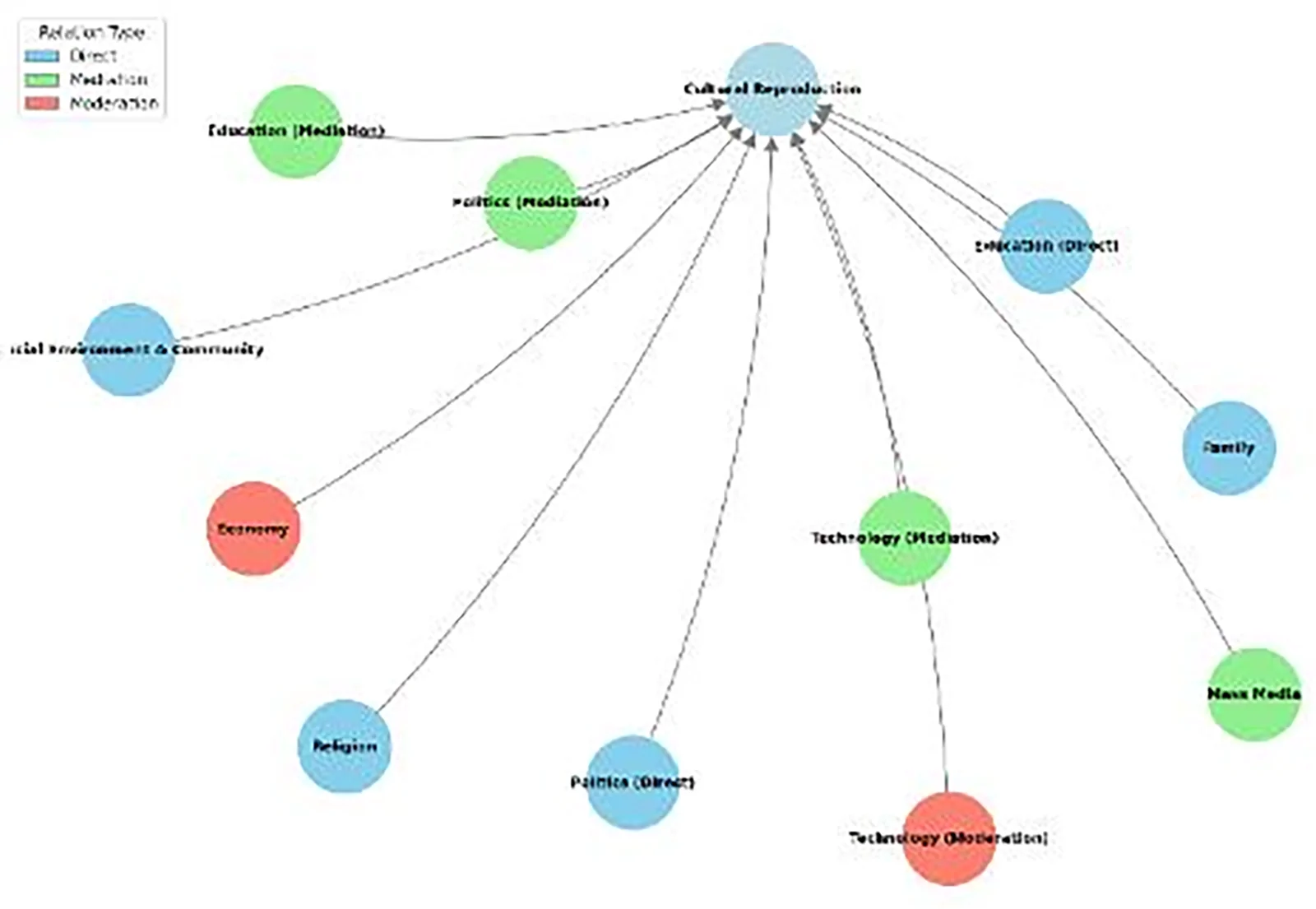
Cultural reproduction model based on ( Jiao et al., 2016) https://zenodo.org/records/18059311.

Cultural reproduction is a complicated process that uses a number of different ways to keep values, norms, and identities the same from one generation to the next. Conceptual diagrams derived from theoretical analysis indicate that social, political, economic, technological, and cultural factors operate not uniformly, but through distinct relationships: direct, mediation, and moderation. The differences in these kinds of relationships show that cultural reproduction happens through interactions at different levels, where microagents, macro institutions, and contextual infrastructures all affect each other. To figure out how culture survives and changes in modern society, we need to understand these different kinds of relationships better.

Family, religion, social community, and certain educational and political roles are directly associated with cultural reproduction. The family, as the principal agent of socialization, transmits habitus, language, and normative values from early childhood ( Bourdieu, 1977). Conversely, religion preserves the continuity of collective moral identity through symbols, rituals, and traditions grounded in quotidian existence ( Geertz, 1973). Social communities create a space for people to interact with each other and work out their shared cultural identities ( Anderson, 1991). Education and politics, in their direct relationship, dictate cultural curricula and policies that shape the propagation of prevailing values ( Bourdieu & Passeron, 1990; Gramsci, 1971). So, direct relationships show that both primary agents and macro institutions are very important for making sure that culture lasts.

Education, mass media, politics, and digital technology are some of the things that can help with mediation. Education serves a dual role: it operates directly and also mediates between the family’s cultural capital and broader social opportunities. Schools not only replicate the classroom structure but also facilitate individual access to social resources ( Bourdieu, 1986). Mass media mediates symbolic representations that shape people’s perceptions of reality, providing material for the construction of identity and behavior ( Kellner, 1995). Politics, via ideology and policy, functions as a conduit that transmits prevailing values into societal consciousness ( Kaufusi, 2024). On the other hand, digital technology speeds up the flow of cultural exchange across borders, making virtual spaces a new place for meaning to be reproduced ( Castells, 2000). This mediation mechanism illustrates that cultural reproduction frequently occurs via intermediary channels that filter, modify, and disseminate specific values.

The moderation factor also plays a big role in controlling how strongly other factors affect things. The main factor that controls things is economics, because the way economic capital is spread out affects how people or groups can get to education, media, and politics. People who have more money are more likely to keep or grow their cultural influence, while people who don’t have much money are often pushed to the side ( Bourdieu, 1986). Digital technology also acts as a moderator, making other factors stronger or weaker depending on how it is used. Digital platforms, for instance, can broaden the accessibility of online education or alter the dynamics of mass media information distribution, simultaneously creating opportunities for counterculture ( Kudelić et al., 2025). Moderation, then, is a contextual variable that affects the size and scope of cultural reproduction.

From the overall analysis, it is evident that cultural reproduction is neither a linear nor a static process; instead, it arises from intricate interactions among various types of relationships. Directly related factors are the main source of cultural value, and mediating factors make sure that those values are spread and changed in society. Moderation, conversely, governs the intensity of influence, allowing cultural reproduction outcomes to differ across social contexts. This pattern aligns with Bourdieu’s (1986) theory of capital, which underscores inter-modal interaction (economic, social, cultural, symbolic) in sustaining social structure, and Gramsci’s (1971) theory of hegemony, which highlights the significance of ideological dominance in perpetuating cultural power. The role of technology and media as mediators and moderators aligns with Castells’ (2000) examination of a networked society, wherein the circulation of digital information constitutes the primary foundation of social reproduction.

This conceptual diagram delineates the factors of cultural reproduction and underscores the necessity of differentiating the types of relationships that operate within the process. The interplay of direct relations, mediation, and moderation demonstrates that cultural reproduction is dialectical, emerging from the interaction among objective structures, subjective agents, and technological and economic contexts. These results indicate the necessity for a comprehensive analytical framework that perceives cultural reproduction as a multi-dimensional and evolving phenomenon. Only through this perspective can the examination of cultural reproduction yield a more comprehensive understanding of how societies sustain and evolve their cultural identities amid global transformation.

## Discussion

### 1. Interpretation of main findings and their relation to research objectives

The results of a systematic review of 143 articles show that cultural reproduction is a complex process that works through multi-level interactions between micro-agents (family, community, religion) and macro structures (education, politics, economics, media, and technology). Overall, these findings confirm that cultural reproduction not only serves to maintain existing social structures but also allows adaptation and transformation of values according to dynamic social contexts. Thus, these results successfully answer the research objectives of analyzing the factors that influence cultural reproduction, explaining the main concepts, and formulating a conceptual model that links them to socio-cultural resilience.

The distribution of factors shows that education, politics, and family are the dominant agents in maintaining cultural sustainability. Education plays a dual role: first, as an instrument of legitimization of dominant values through curriculum and pedagogy (direct reproduction); Second, as a mediator between family cultural capital and social opportunities (indirect reproduction). Politics and economics work to regulate the distribution of capital that determines access to cultural resources, while media and digital technology are becoming new links in the transmission of values across geographical and generational boundaries. Thus, the cultural reproduction model formed is integrative-adaptive, combining direct relationships, mediation, and moderation among the eight main factors. These findings strengthen the position of the research as a theoretical and empirical synthesis that bridges various disciplinary domains in understanding socio-cultural resilience.

### 2. Comparison with previous literature

a. Alignment with Previous Research
  The findings of this study are consistent with the classical theoretical framework of Bourdieu (1977, 1986) which emphasizes the role of education and the family in the inheritance of cultural capital. Schools remain the dominant arena that reproduces the habitus of the middle class through the symbolic legitimacy of the curriculum. Harmony is also seen with Gramsci’s (1971) view of hegemony, in which ideology and politics function to maintain class dominance through social consent, not coercion. In the contemporary context, these results support the findings of Janssen et al. (2024) that cultural reproduction now takes place also in digital spaces, where algorithms and online networks act as new symbolic mediators.
  The study of Ghahramani et al. (2020) and Lv et al. (2025) also strengthens the evidence that cultural heritage and community participation contribute directly to social resilience, especially when integrated with participatory governance and sustainable tourism. Thus, the results of this systematic review confirm the causal relationship between cultural reproduction and socio-cultural resilience, as indicated in the cross-contextual empirical literature.
b. Differences and Contradictions with Literature
  Although in line with classical theory, the results of this study also show an important deviation from Bourdieu’s deterministic approach. In many contemporary studies, it has been found that cultural reproduction not only maintains inequality, but also opens up space for social mobility, as argued by DiMaggio (1982) and Blaskó (2003). The results of the synthesis suggest that individuals with lower class backgrounds can gain mobility through intensification of cultural participation, especially in digital contexts and non-formal education. This means that cultural capital no longer functions exclusively as a tool of domination, but also as a transformative means in the modern context.
  Another difference arises in the role of technology. While Castells (2000) sees technology as a neutral structural mediator, the results of this study found that technology can actually function as a moderator, strengthening or weakening the effects of other factors. For example, high digital access enlarges the reproduction range of cultural values, but digital inequality can widen the gap in cultural participation. It expands the framework of Castells’ network theory by adding a dimension of social amplification in the process of cultural reproduction.
c. New Contributions to Literature
  The main contribution of this research lies in the formulation of an integrative model that combines direct–mediation–moderation mechanisms between factors, resulting in a multi-level understanding of cultural reproduction. This model has not been found explicitly in previous studies, which tend to focus on a single domain such as education or media. In addition, this study introduces a systemic approach to socio-cultural resilience, which sees cultural reproduction not only as a mechanism of conservation, but also as an instrument of adaptation to social, political, and ecological change. This synthesis expands the scope of reproductive theory by adding a dimension of community resilience, which is relevant to the formulation of sustainable development policies and strategies.

### 3. Thematic synthesis of each research question

a. Main Concepts of Cultural Reproduction
  The literature synthesis shows that cultural reproduction is a mechanism of inheritance of values and norms that works through symbolic structures, social institutions, and everyday interactions. Bourdieu’s concepts of habitus, cultural capital, and symbolic violence remain the main foundations. However, contemporary contexts extend it through the digital, participatory, and intersectionality dimensions of identity ( Crenshaw, 1991). Cultural reproduction now takes place not only through education and family, but also through digital platforms that determine symbolic visibility and legitimacy. Therefore, the reproduction of modern culture can be understood as a dialectical process between conservation and innovation in the value system of society.
b. Factors Affecting Cultural Reproduction
  The systematic analysis identified eight main factors: family, education, politics, economy, religion, mass media, technology, and the social-community environment. Education occupies the most dominant position (17.6%), followed by politics (15.3%) and family (14.1%). Each factor has a different type of relationship: direct, mediated, or moderate. Micro factors such as family and religion work directly, while macro factors such as politics, media, and economics function through intermediaries or arrangements of the intensity of influence. This synthesis suggests that cultural reproduction cannot be understood linearly but as a social ecological system that influences each other.
c. Relationships between Factors
  The findings suggest that the interaction between factors forms a dynamic network. Education is the main node that connects the family’s cultural capital with economic and political opportunities. Media and technology mediate the spread of cultural symbols across regions, while economics and technology serve as moderators that determine the scale and extent of the influence of other factors. This pattern reinforces Bourdieu’s (1986) theory of compound capital while complementing Castells’ (2000) network theory by adding a normative layer. Cultural reproduction is thus a relational process that moves through interdependence between social factors.
d. Cultural Reproduction Model as a Form of Socio-Cultural Resilience
  The model developed illustrates that cultural reproduction contributes directly to socio-cultural resilience through three mechanisms: (1) preservation of core community values, (2) adaptation to social and technological change, and (3) symbolic legitimacy of collective identity. Socio-cultural resilience is formed when cultural values and practices are able to maintain social cohesion while adapting to external dynamics such as globalization or environmental crises. This model asserts that resilience does not depend on conservation alone, but on the ability of communities to integrate tradition with innovation.

### 4. Theoretical, practical, and policy implications

a. Theoretical Implications
  These findings enrich Bourdieu’s theory by showing that cultural capital can be transformative, not merely reproductive. The multi-level approach opens space for a synthesis between the theory of cultural capital, Gramscian hegemony, and Castells social networks. Thus, the theory of cultural reproduction developed into a theory of cultural ecology, which views social resilience as the result of systemic interactions between structures, agents, and technologies. Another theoretical implication is the need to expand the definition of cultural capital into the form of digital capital and participatory capital, in line with the social transformation of the 21st century.
b. Practical Implications
  Practically, the results of this study provide a basis for designing adaptive cultural preservation programs. Education must be directed at the transmission of values across generations through the integration of a curriculum based on local wisdom and digital literacy. Governments and cultural institutions can use this model to strengthen community participation in the preservation of intangible cultural heritage (ICH) as advocated by McDermott (2024) and Sun et al. (2024). At the community level, this model can be applied to increase cultural awareness through interactive activities based on digital media, which combine tradition with technological innovation.
c. Policy Implications
  For policymakers, these findings underscore the importance of participatory governance and cross-sectoral collaboration. Cultural preservation policies cannot be top-down but need to be designed as an adaptive cultural policy ecosystem that connects education, the creative economy, and digital technologies. In addition, socio-cultural resilience indicators need to be developed by considering the dimensions of community participation, equity of access to cultural resources, and environmental sustainability. Thus, the results of this study support UNESCO’s policy direction in strengthening the role of cultural heritage as a source of global resilience.

### 5. Unexpected findings and explanations

One unexpected result is the emergence of the ambivalent role of digital technology in the process of cultural reproduction. Although it is expected to be a means of democratizing culture, the results of the synthesis suggest that technology can also reinforce inequality through algorithms that focus on dominant cultures. This phenomenon explains how social media can reproduce symbolic hegemony in a new format. But on the other hand, the digital space also opens opportunities for minority cultures to express their identities and build a global solidarity network. This ambivalence confirms that digitalization is not just an instrument, but a new ideological arena that needs to be critically examined.

In addition, another interesting finding is the role of local communities that has tended to be overlooked in previous literature. Community turns out to play a strategic function in maintaining a balance between value conservation and social adaptation. The practice of cooperation, rituals, and traditional arts are a source of moral strength that sustains social resilience amid modernization pressure. This shows that resilience is not only built through macro policies, but also through micro-social networks that live in people’s daily lives.

### 6. Methodological strengths and limitations

The Systematic Literature Review (SLR) approach is a major strength of this research because it allows for cross-disciplinary integration in a transparent and replicative manner. The use of the PRISMA protocol and MMAT tool ensures the validity of the selection and the reliability of the interpretation of the results. Thematic analysis using NVivo deepens the understanding of the relationships between factors and enables the development of evidence-based conceptual models. However, there are some limitations. First, the dominance of English-language articles has the potential to exclude local non-English literature that is relevant to Asian or African contexts. Second, the analysis only covers the 2015–2025 range, so it does not fully capture the historical dynamics of long-term cultural reproduction. Third, although SLR provides a strong theoretical synthesis, this study does not include direct empirical validation through field observations or in-depth interviews. Therefore, the results are conceptual and need to be tested in a specific empirical context.

### 7. Knowledge gap and direction of advanced research

This study reveals several gaps in knowledge that open up opportunities for further study. First, there is still limited research on the reproduction of digital culture in developing countries, even though online space is now the main arena for cultural interaction. Second, there is a need for cross-generational empirical studies that observe the transmission of cultural values from families to digital communities, including the dynamics of resistance and identity hybridization. Third, the integration between cultural reproduction and environmental resilience is still minimal, even though climate change significantly affects local cultural practices.

To fill these gaps, future research can combine mixed-methods with a community-based participatory approach. This approach will allow for empirical validation of the developed model while enriching the theory with contextual data. Interdisciplinary studies between cultural sociology, communication technology, and policy studies also need to be expanded to understand new dynamics in global cultural reproduction.

This discussion emphasized that cultural reproduction is the main foundation of socio-cultural resilience. Through the complex interaction between micro-agents and macro structures, cultural values and identities are not only maintained but also adapted in the face of global change. This research expands on classical theory by adding digital, participatory, and social ecology dimensions that make cultural reproduction a mechanism of collective resilience. With an integrative model that combines direct relationships, mediation, and moderation, the results of this study make new contributions to the theory, practice, and policy of cultural preservation. Through this synthesis, cultural reproduction is understood not only as a process of preserving the past, but as a future adaptive strategy for society to survive and develop in the ongoing flow of globalization and technological transformation.

This section should be completed as per the PRISMA checklist, item 23. (For scoping reviews, see PRISMA-Scr, items 19-21).

## Conclusion

This study, derived from a systematic synthesis of 143 studies, affirms that cultural reproduction is a multidimensional process characterized by intricate interactions between micro-agents—such as family, community, and religion—and macro structures—such as education, politics, economics, media, and technology. The findings indicate that cultural reproduction serves not only as a means of preserving values but also as an adaptive strategy for enhancing the socio-cultural resilience of communities. The integrative model created brings together eight key factors that interact with each other in direct, mediated, and moderated ways. These factors work together to create a cultural reproductive ecosystem that can change with social, political, and technological changes. This study enhances Bourdieu’s theory by incorporating digital, participatory, and social ecology dimensions, while also broadening the comprehension of cultural capital as a transformative rather than merely reproductive tool. This theoretical contribution strengthens the research’s role as a link between the traditional theory of cultural reproduction and the modern resilience approach, which views culture as the basis of social sustainability and collective identity. In practice, these results serve as a foundation for the formulation of community-oriented cultural preservation policies, the incorporation of local values into education, and the utilization of digital technologies to enhance inclusive cultural access and participation.

### Suggestion

This study acknowledges methodological constraints, including the predominance of English-language literature, the restricted analysis period (2015–2025), and the absence of direct empirical validation for the developed conceptual model. Consequently, additional research is advised to implement a mixed-methods approach, incorporating field observation and participatory interviews, to evaluate the contextual validity of the model. Cross-generational and cross-cultural studies are essential for comprehending the dynamics of cultural value transmission in the digital era, particularly within non-Western societies that remain under-represented in the literature. Furthermore, subsequent research must broaden its emphasis on the amalgamation of cultural reproduction with environmental resilience and the sustainability of the creative economy, considering its significance amid global crises and climate change. The findings of this study highlight the imperative for policymakers to establish a participatory and collaborative cultural policy ecosystem that integrates education, technology, the creative economy, and the safeguarding of intangible cultural heritage. So, cultural reproduction can be a way to keep your identity and a group strategy for making society more resilient in the long run.

## Data Availability

No data associated with this article. Repository name: Dataset Article SLR: Models Of Cultural Reproduction As Drivers Of Socio-Cultural Resilience: Insights From A Systematic Review https://zenodo.org/records/18058118 and https://zenodo.org/records/18059311 ( Al Faqi, et al., 2025). This project contains the following extended data:
1. Dataset Selection Process (Collection of article data used for literature review) 2. PRISMA 2020 Checklist 3. Dataset Graphic Figure 2 4.
Figures 1, 2, 3 and 4 Repository name: PRISMA checklist for Models Of Cultural Reproduction As Drivers Of Socio-Cultural Resilience: Insights From A Systematic Review https://zenodo.org/records/18058118 (Al Faqi, et al., 2025). Data are available under the terms of the Creative Commons Attribution 4.0 International license (CC-BY 4.0).
