# Models of Cultural Reproduction as Drivers of Socio-cultural Resilience: Insights from a Systematic Review

**DOI:** 10.12688/f1000research.174513.2

**Published:** 2026-05-25

**Authors:** Mifdal Zusron Alfaqi, Moh Fadli, Alfi Haris Wanto, Tjahjanulin Domai, Akil Fitra Sholakodin

**Affiliations:** 1 Doctoral Program in National Resilience Science, Universitas Brawijaya, Malang, East Java, 65145, Indonesia; 2 Constitutional Law, Universitas Brawijaya, Malang, East Java, 65154, Indonesia; 3 Public Administration Science, Universitas Brawijaya Fakultas Ilmu Administrasi, Malang, East Java, 65145, Indonesia; 4 Sociology, Universitas Indonesia Fakultas Ilmu Sosial dan Ilmu Politik, Depok, West Java, 16424, Indonesia

**Keywords:** cultural reproduction, socio-cultural resilience, cultural capital, digitalization, social systems.

## Abstract

**Aim:**

To examine the determinants influencing cultural reproduction, comprehend the fundamental concepts of cultural reproduction, and evaluate the model of cultural reproduction as a manifestation of socio-cultural resilience.

**Materials and Methods:**

The research employed the Systematic Literature Review (SLR) methodology, utilizing the PRISMA protocol and the PICO framework. Data were sourced from Scopus, Web of Science, ProQuest, and Google Scholar, chosen based on inclusion criteria (2015–2025, empirical articles, pertinent to socio-cultural resilience). Of the 688 initial articles, 143 studies met the criteria. The Mixed Methods Appraisal Tool (MMAT) was used to assess quality, and NVivo 14 was used for thematic synthesis to find patterns of direct relationships, mediation, and moderation between factors.

**Results and discussions:**

The analysis identified eight principal factors: education, politics, family, economy, media, technology, religion, and community. Politics (15.3%) and education (17.6%) were the most important factors. Cultural reproduction operates through micro–macro interactions involving direct relationships (family, religion, community), mediation (education, media, politics, technology), and moderation (economics, technology). The resulting integrative model elucidates how this amalgamation of factors enhances socio-cultural resilience through conservation and value adaptation.

**Conclusions:**

Cultural reproduction serves both as a means of value preservation and as an adaptive mechanism that enhances social resilience. This research enhances Bourdieu’s theory by incorporating digital and participatory dimensions, providing a conceptual foundation for sustainable cultural policy.

## Introduction

The erosion of cultural diversity, evidenced by the looming extinction of nearly half the world’s 7,000 languages, signifies a profound crisis in global socio-cultural stability. Due to globalization, urbanization, and socio-political pressures, linguistic and cultural diversity is forecast to experience a swift reduction across various continents ( Bromham et al., 2022). While modern global shifts have altered how communities engage with their heritage, indigenous identity is increasingly threatened by global cultural homogenization, which marginalizes local knowledge systems. This pressure creates a form of “epistemic domination,” where global power dynamics shape knowledge hierarchies that legitimize Western epistemologies while suppressing indigenous and local narratives (Mohan et al., 2026; Sánchez-Vera, 2025). These dominant frameworks influence cultural identities by promoting modernity narratives that often sideline rural and indigenous perspectives. This shift, compounded by the challenges of climate change and conflict, necessitates a deeper examination of cultural reproduction as a fundamental mechanism for building socio-cultural resilience and reclaiming cultural sovereignty by resisting such global power dynamics.

The collapse of intergenerational transmission mechanisms represents more than a loss of heritage; it creates a vacuum in community adaptive capacity and social cohesion. Current discourse increasingly positions Intangible Cultural Heritage (ICH) as a functional asset in resilience strategies, yet its efficacy depends heavily on the synergy between indigenous agency and formal policy backing. Rather than relying solely on digital archives, cultural vitality is sustained through community-centered approaches that foster traditions through direct apprenticeship and embodied skill transmission. Within a socio-technical resilience framework, these localized practices help ICH systems adapt to change by fostering sociomaterial structures and anticipatory practices that allow traditions to evolve without losing their essence (Amir & Kant, 2018; Gersonius et al., 2012). McDermott and Craith (2024) contends that intangible cultural heritage (ICH) facilitates climate change adaptation by incorporating cultural practices into resilience strategies. Without a model that bridges these domains, socio-cultural vulnerabilities are anticipated to escalate.

While classical theories of cultural reproduction, notably Bourdieu’s, provide a foundation for understanding the persistence of social structures, they are criticized for being overly deterministic. Critics argue that his framework, particularly the concept of habitus, tends to collapse agency into structure, creating a “pessimism of reason” in which social fields appear rigid and resistant to change (Felipe Miguel, 2015; Miguel, 2015; Paludo, 2026). Such theories often underestimate the role of collective agency and transformative moments in disrupting reproduction (Fowler, 2020; G. Sapiro, 2025; V. Sapiro, 2014). Several empirical studies have endeavoured to address this question.

Ghahramani et al. (2020) found that cultural heritage serves as a source of resilience when linked to community-based participatory governance, while Lv et al. (2025) demonstrated that intangible cultural heritage (ICH) can facilitate sustainable development through dynamic reciprocal relationships. However, a significant research gap persists; most studies remain localized, sector-specific, or focused on a single variable. Furthermore, the current literature lacks a systematic framework for categorizing these elements as mediators or moderators, leaving a notable absence of in-depth analysis of causal mechanisms, such as the interplay between material practices and indigenous agency that facilitate cultural adaptation. Existing work often overlooks how contextual variability, including cultural and socio-economic nuances, shapes resilience dynamics. This fragmentation highlights the need for a holistic model that integrates multiple dimensions of cultural reproduction to elucidate causal relationships in diverse and volatile settings.

This study seeks to examine the determinants influencing cultural reproduction, elucidate its fundamental concepts, and formulate an adaptive-integrative paradigm of cultural reproduction as a manifestation of socio-cultural resilience. This research addresses a critical gap in the literature by providing an integrative framework that links cultural practices, social structures, and heritage policy. Unlike previous fragmented studies, this paradigm offers a multidimensional lens for understanding how communities exercise indigenous agency to navigate structural constraints and global disruptions. Furthermore, this research offers practical strategic insights for policy formulation, particularly in the areas of intangible cultural heritage (ICH) conservation, participatory governance design, and the development of resilience indicators. By providing a comprehensive conceptualization of how cultural values are preserved and adapted, this study enhances the understanding of socio-cultural resilience amid global transformations. Ultimately, by providing a systematic categorization of mediating and moderating factors, this research offers a robust framework for fostering cultural sovereignty and resisting epistemic domination in diverse and volatile settings.

## Methods

The methodology of this study employs the Systematic Literature Review (SLR) to synthesize exicting knowledge on cultural resilience. Adhering to the PRISMA (Preferred Reporting Items for Sytematic Reviews and Meta Analyses) guidelines. This method ensures a transparent, replicable, and rigorous process for identifying, selecting, and critically appraising relevant research. SLR enables researchers to identify, assess, and synthesize pertinent evidence in a clear and reproducible manner ( Snyder et al., 2024). Consequently, this methodology is suitable for addressing the research deficiencies identified in the introduction and for providing a comprehensive understanding across various domains. This study employs the Population, Intervention, Comparison, Outcome (PICO) framework to elucidate the SLR framework and to formulate research questions. The following Table 1 shows the details:

**Table 1.** PICO analysis framework.

Component	Description
Population	Communities and social groups involved in cultural reproduction practices, both local and transnational
Intervention	Mechanisms, strategies, or policies that affect cultural reproduction (intergenerational transmission, digital participation, cultural heritage policies)
Comparison	Differences in geographical, temporal, or sectoral contexts in the application of cultural reproduction
Outcome	Socio-cultural resilience, social cohesion, and adaptive capacity of communities

The literature search strategy is systematically designed using several major academic databases, namely Scopus, Web of Science, ProQuest, and Google Scholar. The search process is carried out with a combination of keywords and Boolean operators as follows:

(“cultural resilience” OR “cultural sustainability” OR “cultural adaptation” OR “cultural preservation”) AND (“community” OR “society” OR “group” OR “population”) AND (“identity” OR “heritage” OR “tradition” OR “values”) AND (“adaptation” OR “response” OR “recovery” OR “transformation”) AND (“disaster” OR “crisis” OR “change” OR “challenge”)

The search results are then exported for further filtering.

Inclusion and exclusion criteria are established to ensure the relevance and quality of the sources used. The following Table 2 provides a breakdown of these criteria:

**Table 2.** Data selection process.

Inclusion criteria	Exclusion criteria
Empirical articles in English	Articles in the form of reviews or non-empirical
Publications in the last 10 years (2015–2025)	Publications under 2015
Focus on cultural reproduction and socio-cultural resilience	Articles that only discuss cultural aspects with no relevance to resilience
Full access (open access or institutional access)	Articles with limited access without full availability

The PRISMA (Preferred Reporting Items for Systematic Reviews and Meta-Analyses) protocol is used to pick the articles so that each step is clear and can be traced ( Page et al., 2021). PRISMA is utilized throughout the identification phase, title and abstract screening, comprehensive text assessment, and the ultimate inclusion of the article. This protocol allows for the replication and methodological evaluation of the research flow. The latest version of the Mixed Methods Appraisal Tool (MMAT) is used to check the quality of articles. This tool lets you compare different methods, including quantitative, qualitative, and mixed methods ( Hong et al., 2018). This evaluation emphasizes the lucidity of the research design, the validity of the instrument, the pertinence of the results, and their alignment with the research objectives. The following Figure 1 provides a breakdown of these PRISMA criteria:

**Figure 1.**
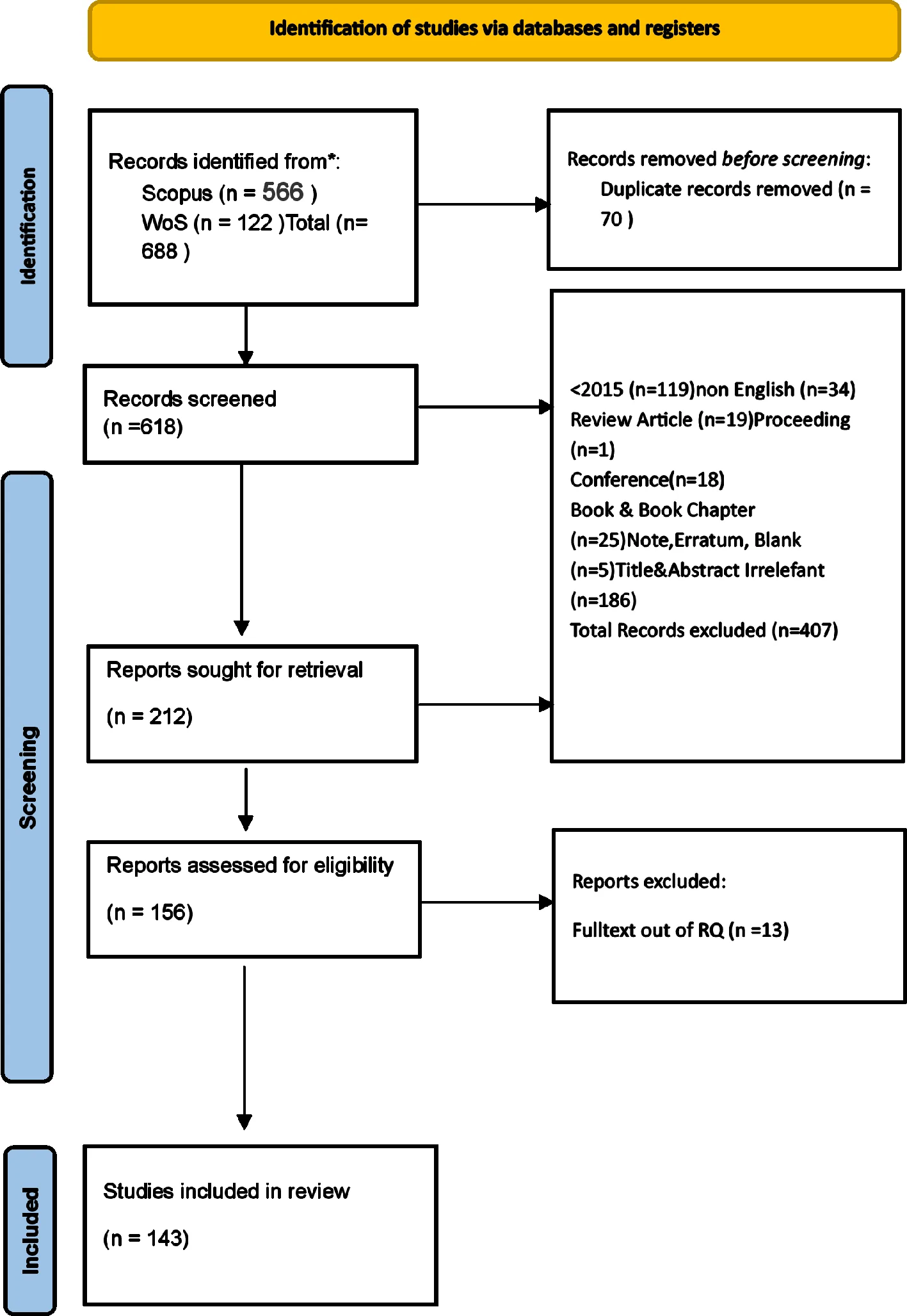
SLR’s PRISMA flows chart visualizing data selection process.

This study’s PRISMA flow diagram shows how articles were chosen in a systematic way that met the study’s criteria. We got a total of 688 articles from the Scopus (566) and WoS (122) databases. However, 70 of them were deleted because they were duplicates, leaving us with 618 articles to choose from. 407 articles were left out of the screening stage because they didn’t meet the criteria, such as being published before 2015, in a language other than English, in the form of review articles, proceedings, conferences, books, notes, or abstract titles that weren’t relevant. After that, 212 reports were looked for to download, but 56 of them could not be found, leaving 156 articles that were fully checked for eligibility. Out of these, 13 articles were removed because the full-text content did not align with the research question. So, 143 studies were finally deemed possible and added to the review. The arrow to the right in the diagram shows the articles that were published at each step of the selection process. This makes it easy to see how the process of identification, screening, and inclusion worked in this systematic review.

Data extraction entails the collection of essential information from each article, encompassing the research objectives, methodology, context, key findings, and implications. After that, NVivo 14 software was used to analyse the data using thematic analysis methods. The process involves initial coding, categorization, identification of principal themes, and thematic synthesis to address research inquiries. This method enables a comprehensive examination of the interrelationships among cultural reproductive factors and their contribution to socio-cultural resilience.

To enhance accuracy and mitigate bias, the study was executed by three researchers with a distinct allocation of responsibilities. The first researcher (P1) is in charge of the first search design and filtering of the article. The second researcher (P2) is in charge of judging the quality of the articles and getting the data out of them. The third researcher (P3) concentrated on data analysis using NVivo and the synthesis of findings. All researchers participated in triangulation discussions to guarantee the consistency of interpretation and the validity of findings. This methodology is anticipated to yield a substantial contribution to addressing research inquiries concerning concepts, factors, interrelationships among factors, and cultural reproduction models as expressions of socio-cultural resilience. A methodical, open, and evidence-based approach lays the groundwork for generating findings that are both academically and practically sound.

## Results

Several studies emphasize the important role of cultural reproduction in increasing socio-cultural resilience ( Ghahramani et al., 2020; Lv et al., 2025; Gramsci, 1971; Bourdieu, 1986; Castells, 2000). This study identifies the main factors that influence cultural reproduction, such as education, politics, family, and technology, as well as how these factors contribute to the adaptation of cultural values in response to dynamic social contexts. Education emerges as the most dominant determinant (17.6%), serving as a primary site for stabilizing social structures through the legitimation of dominant cultural capital ( Bourdieu & Passeron, 1990). However, the findings suggest that the curriculum is now being contested as an arena for pedagogical struggle, where indigenous values are reintegrated to foster long-term resilience. Likewise, politics and the family have a significant impact, with politics shaping cultural discourse through state policies and ideologies ( Gramsci, 1971), while the family serves as the main agent in transmitting cultural norms and values between generations ( Bourdieu, 1977). While digital technologies are traditionally seen as mediators of global cultural transmission ( Castells, 2000), contemporary literature increasingly frames them as tools for indigenous agency. These platforms allow communities to navigate structural constraints and foster cultural sovereignty, ensuring that adaptation does not lead to the loss of core identity. These findings confirm that cultural reproduction is not a static process but rather develops through the interaction of micro and macro factors, with each factor playing a unique role in strengthening social resilience. Figure 2 presents the percentage of analyzed articles on cultural reproduction.

**Figure 2.**
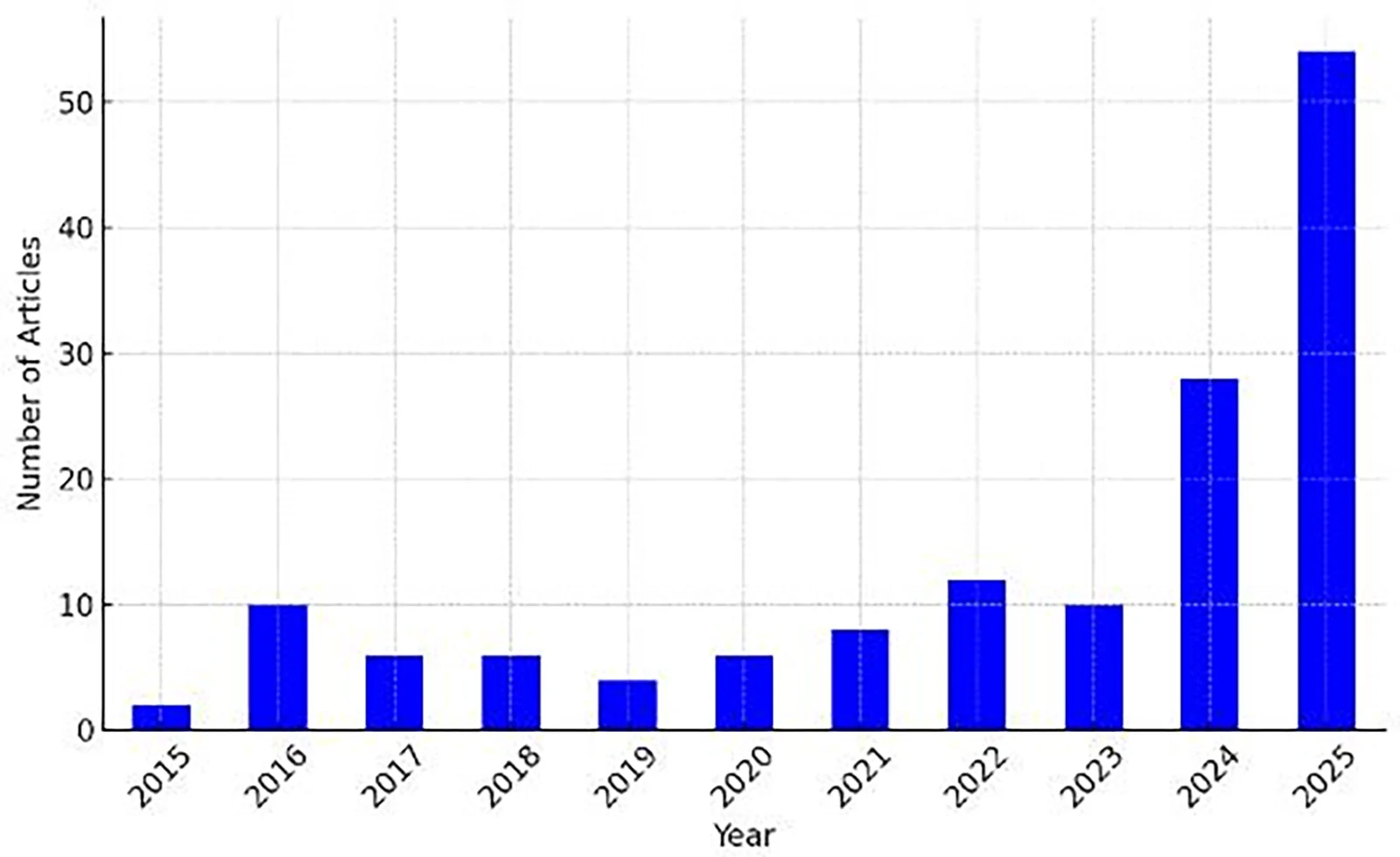
Percentage of articles analyzed.

The progressive growth in publications since 2015 ( Figure 2) indicates a significant shift in the academic paradigm where cultural reproduction is no longer viewed as a static inheritance, but as a dynamic strategic response to global instability. This upward trajectory suggests that scholarly discourse has evolved in tandem with global disruptions, forcing a re-evaluation of how communities maintain identity through an adaptive habitus. The number of articles was still low in the first few years after 2015. After that, the graph showed a steady rise, indicating that research was becoming more intense and that issues in the academic world were becoming more important. Researchers may pay closer attention to years with peaks in specific areas. These peaks may be due to changes in theory, policy, or global events. In the meantime, small year-to-year changes show how the research focus has shifted and how the number of publications in the database has changed ( Yerlanova et al., 2025). Overall, this graph shows a positive trend, with more recent publications on the rise. This shows that research topics are still an important part of modern scientific discourse. Figure 3 shows the novelty of the studies analyzed in the VosViewer application.

**Figure 3.**
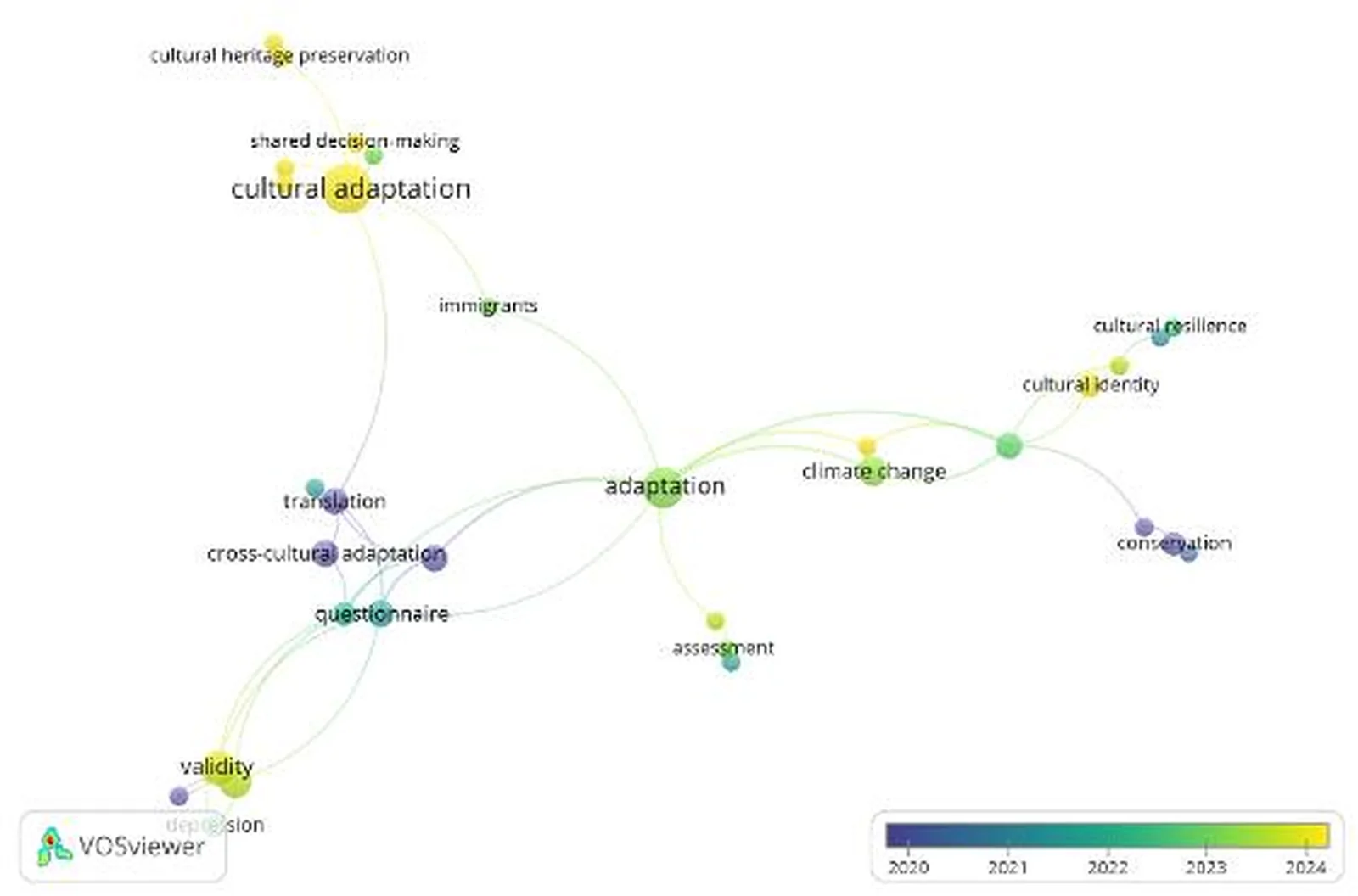
Changing trends in research topics on Cultural Reproduction.

The VOSviewer overlay visualization ( Figure 3) captures a critical conceptual evolution. The temporal pattern illustrates a transition from methodological concerns in 2020 toward a multidisciplinary focus on cultural resilience and shared decision-making by 2024. This shift signifies that scholarly discourse has moved beyond purely technical validation to address how communities exercise agency to maintain identity amid climate change and social disruption ( McDermott & Craith, 2024). The central term “adaptation” serves as the principal nexus, extending to diverse domains, including cultural adaptation, climate change, assessment, and cultural identity. The temporal pattern shows that the first study (2020–2021, purple-blue) focused more on methodological issues such as translation, questionnaires, and validity. As we move into the 2022 (green) period, people are more interested in social and environmental issues such as climate change, immigration, and conservation. The most recent trend (2023–2024, in yellow) focuses on the cultural aspect, especially cultural adaptation, cultural resilience, cultural heritage preservation, and shared decision-making. This shows that research is moving away from a technical approach and toward a multidisciplinary understanding of identity, sustainability, and community participation. The transition from methodological clusters in 2020 to “cultural resilience” and “shared decision-making” in 2024 signifies a critical evolution. This suggests that scholarly discourse has shifted from purely technical validation to addressing how communities maintain identity amid climate change and social disruption. This pattern illustrates the progression of the theme from a conceptual framework to the implementation of adaptation within a more expansive cultural and environmental context.

### Cultural Reproductive Factors

Factors that influence cultural reproduction span a wide range of dimensions, including education, politics, economics, family, media, religion, technology, and social community. Each factor plays a role in maintaining, transmitting, and transforming cultural values, norms, and identities across generations. Education and politics occupy a dominant position due to their roles in shaping social structure and legitimacy, while other factors serve as complementary arenas of support. A comprehensive picture of the distribution of these factors is presented in Table 3.

**Table 3.** Distribution of keywords based on cultural reproduction factors.

Factor	Description	Verbatim quotes	Number of relevant citations	List of citations
Family	A primary socialization agent who instills cultural values, norms, languages, and practices from an early age.	“The family is the primary site where cultural capital is transmitted and embodied” ( Bourdieu, 1977, p. 87).	12	( Alhajj et al., 2017; Chrzan-Rodak et al., 2024; Deffner & McElreath, 2022; Sémah, 2017)
Education	Schools reproduce the habitus and legitimacy of the dominant culture through pedagogical curricula and practices.	“Education systems contribute to the reproduction of the structure of power relations and symbolic relations between classes” ( Bourdieu & Passeron, 1990, p. 54).	15	( Alhajj et al., 2017; Biben et al., 2025; Lui & Johnston, 2019; Pardoel et al., 2022)
Mass Media	The media disseminates representations, symbols, and ideologies that defend or challenge hegemony.	“Media culture provides the materials for constructing views of the world, behavior, and identities” ( Kellner, 1995, p. 1).	10	( Binion et al., 2024; Coffey & Noble, 1996; W. Liu, 2017; Qamar & Ibrahim, 2024; Sultan et al., 2024)
Religion	Religion nurtures rituals, morality, and collective identities that are passed down through generations.	“Religion is a system of symbols which acts to establish powerful, pervasive, and long-lasting moods and motivations” ( Geertz, 1973, p. 90).	8	( Geertz, 1981; Anwar et al., 2025; Kline et al., 2018; Ragab et al., 2021; Seabra et al., 2025; Zhu & Feng, 2024)
Economics	Economic structure and capital distribution affect access to cultural resources.	“The forms of capital … are at the root of the reproduction of social hierarchies” ( Bourdieu, 1986, p. 241).	11	( Casciani et al., 2022; Fanari & Kim, 2025; Gibson et al., 2025; Kudelić et al., 2025; Paplikar et al., 2022; Pascoe & McLeod, 2016)
Politics and Power	The state and political institutions influence cultural reproduction through ideology, laws, and policies.	“The supremacy of a social group manifests itself in two ways, as ‘domination’ and as ‘intellectual and moral leadership’” ( Gramsci, 1971, p. 57).	13	( Chiang & Chang, 2018; Deffner & McElreath, 2022; Huang et al., 2025; Martins & Sá, 2025; Yerlanova et al., 2025)
Technology and Digitalization	Digital platforms accelerate cultural reproduction through algorithms and global networks.	“The network society is a social structure based on networks activated by digital information and communication technologies” ( Castells, 2000, p. 21).	9	( Koyuncu et al., 2020; Nur et al., 2025; Qamar & Ibrahim, 2024; Self et al., 2022; Sultan et al., 2024)
Social Environment and Community	Interaction within the community creates a social space for cultural reproduction and transformation.	“Communities are to be distinguished, not by their falsity/genuineness, but by the style in which they are imagined” ( Anderson, 1991, p. 6).	7	( Kuzmanov, 2025; Lu & Phanlukthao, 2025; Pagán et al., 2025)

The study of donut tables and diagrams reveals the allocation of literary focus on eight primary factors that affect cultural reproduction. The study confirms the enduring dominance of formal institutions in cultural transmissions, with Education appearing in 17/6% of the literature. This suggests that despite the rise of digital spaces, the classroom remains the primary site for reproducing “habitus”. Education serves as a formal teaching medium and a mechanism for reproducing social habitus that sustains class structures and social hierarchies ( Bourdieu & Passeron, 1990). The curriculum, language of instruction, and pedagogical methods systematically reinforce the legitimacy of dominant cultures, rendering Education a strategic domain for perpetuating or contesting social inequality. The curriculum can serve as a bridge between families’ cultural capital and broader social opportunities. By embedding inclusive pedagogical practices, such as dialogic Education and participatory approaches, educators can challenge traditional power dynamics and create pathways for marginalized groups to access social mobility (Chen et al., 2025; Ordem, 2023).

Thirteen citations, or 15.3%, put political and power factors in second place. This demonstrates the significance of the state and political institutions in influencing the trajectory of cultural reproduction via policies, regulations, and ideologies. Gramsci (1971) elucidates that political hegemony is executed not solely through coercive means, but also via ideological domination that compels subordinate groups to perceive dominant values as inherently natural. Political hegemony often legitimizes itself by controlling cultural narratives and discourses, making its ideology appear as common sense or the natural order of things. This symbolic power reinforces the dominant group’s position while marginalizing alternative perspectives (Čufar & Hawlina, 2025; Giroux, 2012). So, politics controls the flow of cultural discourse by granting it symbolic legitimacy that is part of society’s collective consciousness.

Third, the family factor accounted for 12 citations, or 14.1%, indicating that the family is the primary socialization agent that passes down language, customs, and cultural values from a young age ( Bourdieu, 1977; Lareau, 2011). Cultural capital conveyed within the family provides a basis for individuals to acquire and accumulate additional forms of capital, both economic and symbolic, in subsequent stages of life. For example, dinner-table conversations in immigrant families facilitate language socialization and heritage preservation, thereby strengthening cultural identity (Wu et al., 2025). The economy ranks fourth, with 11 citations (12.9%). This shows that the distribution of economic capital has a significant effect on how culture reproduces itself. Families with greater economic resources can provide better educational opportunities, thereby enhancing the accumulation of cultural and social capital. For example, parental education and economic circumstances are strongly linked to children’s mental health and social mobilit (Dowling, 2016; Jeong & Veenstra, 2017). Conversely, economic constraints in low-income families limit their ability to pursue cultural ideals, such as healthy or ethical eating, despite their cultural knowledge (Beagan et al., 2016). Bourdieu (1986) asserted that the allocation of economic capital is intricately linked to cultural capital, as access to education, media, and political arenas is significantly influenced by the availability of economic resources.

The mass media factor accounted for 10 citations, or 11.8%, indicating that the media is an important means of spreading cultural ideas, ideologies, and group stories. The media is not impartial; it is a battleground for meaning where prevailing forces strive to maintain hegemony, while opposing forces endeavor to contest it ( Kellner, 1995). Media plays a critical role in constructing and maintaining hegemony, defined as the dominant group’s ability to secure consent from subordinate groups through ideological leadership rather than coercion (Özer, 2011). Also, technology and digitalization are at 9 citations, or 10.6%, which shows how information technology has changed the way culture is passed down from one generation to the next in the modern world ( Castells, 2000). Digital platforms, algorithms, and transnational networks facilitate the transcendence of cultural boundaries, engendering novel modes of interaction that frequently result in identity hybridization. Younger generations, particularly Millennials and Generation Z, have integrated digital tools into their daily lives, making technology a core aspect of cultural consumption and communication. This shift has simplified cross-cultural interactions and bridged generational divides (Kshirsagar & Ingle, 2025; Lifintsev & Wellbrock, 2019).

The religious factor accounted for 8 citations, or 9.4%, indicating that, even as society changes quickly, religion remains the primary means of passing down moral values and a sense of community. Geertz (1973) says that religion’s symbolic system creates meanings that shape the collective consciousness through rituals, traditions, and moral teachings. Religious rituals, such as communal assemblies and sacrificial practices, foster group cohesion, moral outlook, and shared values (Antonello, 2017; Yang et al., 2025). Lastly, the social and community environment, with 7 citations (8.2%), demonstrated that daily interactions within local communities, ethnic groups, and social spaces are crucial for sustaining the dynamics of cultural reproduction. The community serves as both a site for conservation and a platform for negotiating meaning and identity, which evolve in response to contextual factors ( Anderson, 1991).

The citation distribution indicates that the literature predominantly focuses on structural factors, including education, politics, and economics, that institutionally influence patterns of cultural reproduction. These factors constitute the predominant focus in academic studies, whereas community and religious factors are comparatively underemphasized. This pattern indicates that research often emphasizes macro mechanisms over macrodynamic, despite the significance of both in cultural reproduction. However, some works argue for integrating micro- and macro-level perspectives to understand better cultural evolution and its spatial or temporal dynamics (Kandler et al., 2012; Kashima, 2014). These results suggest that comprehending cultural reproduction necessitates a holistic approach that examines both substantial institutions and quotidian social interactions.

### Types of factor relationships with cultural reproduction

The types of relationships between factors and cultural reproduction show a diversity of mechanisms at work, ranging from direct relationships such as family, religion, and community to mediations run by education, media, politics, and technology. In addition, economics and technology play a moderating role in strengthening or weakening the influence of other factors. This pattern shows that cultural reproduction does not occur in isolation, but rather through a combination of micro and macro mechanisms that complement each other. A comprehensive overview of this type of relationship is presented in Table 4.

**Table 4.** Distribution keyword based on Factor Relationships.

Factor	Types of relationships	Description	Verbatim quotes	Citation
Family	Immediately	The family is the primary socialization agent that directly transmits values, norms, language, and habitus to the next generation.	"The family is the primary site where cultural capital is transmitted and embodied"	( Qamar & Ibrahim, 2024; Saisuk et al., 2025)
Education	Immediately	Education directly reproduces the habitus and legitimacy of the dominant culture through pedagogical curriculum and practices.	"Education systems contribute to the reproduction of the structure of power relations and symbolic relations between classes"	( Casciani et al., 2022; Grarup et al., 2019; Monroe et al., 2024; Nittas et al., 2024)
Education	Mediation	Education also acts as a mediator between the family's cultural capital and wider social opportunities.	"Schools mediate the transmission of cultural capital across generations"	( Datzmann et al., 2021; Naralieva et al., 2025; Nittas et al., 2024)
Mass Media	Mediation	The media serves as a mediator in the dissemination of cultural symbols, discourses, and representations that form the collective consciousness.	"Media culture provides the materials for constructing views of the world, behavior, and identities"	( Correa & Inamdar, n.d.; Naralieva et al., 2025; Zhao et al., 2025)
Religion	Immediately	Religion directly maintains its identity and moral values through rituals, symbols, and traditions.	"Religion is a system of symbols which acts to establish powerful, pervasive, and long-lasting moods and motivations"	( Glasbey et al., 2023; Suhodolli, n.d.; Tsfati et al., 2023)
Economics	Moderation	The distribution of economic capital does not directly create cultural reproduction, but moderates individual or group access to education, media, and other cultural resources.	"The forms of capital … are at the root of the reproduction of social hierarchies"	( Binder et al., 2025; P. Liu, 2025; Tahara et al., 2023; Yamaguchi & Mitsuhashi, 2025)
Politics and Power	Immediately	Politics shapes the reproduction of culture directly through state policies and institutions.	"The supremacy of a social group manifests itself in two ways, as 'domination' and as 'intellectual and moral leadership'"	( Binion et al., 2024; Holdar et al., 2021; Kasemets & Nugin, 2022; Lu & Phanlukthao, 2025)
Politics and Power	Mediation	Politics also mediates the legitimacy of dominant values into public consciousness through ideology.	"Ideology functions to secure the spontaneous consent of the masses"	( Biben et al., 2025; Nur et al., 2025; Self et al., 2022)
Technology and Digitalization	Mediation	Technology is a mediator in accelerating the flow of culture across borders.	"The network society is a social structure based on networks activated by digital information and communication technologies"	( Aydin et al., 2025; Gibson et al., 2025; McCarty et al., 2024; Wang & Nam, 2025)
Technology and Digitalization	Moderation	Technology also acts as a moderator that reinforces or weakens the influence of other factors.	"Digital platforms can amplify or constrain cultural flows"	( Aksoy, 2024; Paplikar et al., 2022; Zhang et al., 2025)
Social Environment and Community	Immediately	Communities directly shape cultural reproduction spaces through everyday interactions, social practices, and collective imagination.	"Communities are to be distinguished, not by their falsity/genuineness, but by the style in which they are imagined"	( Janssens et al., 2017; Kaufusi, 2024; Policastro et al., 2019; So & Ip, 2020; Tverskoi et al., 2024)

The results of the table and heatmap study show that cultural reproductive factors have different types of relationships, reflecting the diversity of mechanisms at work in the process of inheriting values, norms, and identities. First, family and religious factors have a direct relationship to cultural reproduction. The family, as the primary socialization agent, instills habitus, language, and norms that are automatically inherited across generations ( Bourdieu, 1986). Families play a central role in cultural reproduction by instilling habitus, language, and norms that are inherited across generations. This process is influenced by structural factors such as family composition, parenting styles, and intergenerational relationships (Harrison, 2014; Munarini & Kgadima, 2023). Similarly, religion reproduces values through symbols, rituals, and belief systems that make up a collective identity ( Geertz, 1981). In this context, the family and religion are fundamental sources of cultural consciousness, without additional intermediaries or mechanisms, since they are integrated into society’s daily practices. Families practicing religion often serve as the primary context for religious socialization, embedding values and norms that persist into adulthood. However, the effectiveness of religious socialization varies across cultural and secular contexts (Legault-Leclair, 2026).

Furthermore, the education factor occupies a dual position, with both a direct relationship and a mediating role. In a direct relationship, education reproduces social structures through curriculum, academic evaluation, and pedagogical practices that instill dominant habitus ( Jiao et al., 2016). However, education also serves as a mediator, bridging the cultural capital inherited from the family into the broader social system. For example, children from families with high cultural capital tend to take advantage of educational opportunities more easily, thereby gaining greater access to social resources. In this sense, education not only reproduces class structures but also fosters social mobility by enabling access to institutional resources and networks, particularly through interactions between parents, teachers, and communities (Natkhov, 2012).

In addition, social and cultural capital developed through education contributes to reducing behavioral problems and delinquency, highlighting its broader social impact (Parcel & Hendrix, 2014). Thus, education not only establishes structure but also mediates the distribution of capital between generations.

Mass media and digital technology factors play a role mainly through mediation relationships. Mass media provide symbolic representations that shape public opinion, direct behavior, and mediate individual perceptions of social reality ( Anwar et al., 2025). Meanwhile, digital technology serves as a mediator that accelerates cultural flows across geographical boundaries, creating new forms of cultural interaction in cyberspace ( Anwar et al., 2025). These two factors confirm that cultural reproduction does not only take place in physical spaces, but also in symbolic and digital spaces that are increasingly dominant in modern society.

In addition to serving as a mediator, digital technology also acts as a moderator. Technology amplifies or weakens the effects of other factors, for example, by expanding the reach of education through online platforms or by changing how mass media distributes information. In other words, technology creates conditions that regulate the intensity of the influence of other factors on cultural reproduction ( So & Ip, 2020). Digital technology has reshaped cultural production, consumption, and dissemination, enabling global communication and cultural exchange. This has led to both cultural enrichment and challenges, such as cultural homogenization, in which dominant cultures overshadow local identities (Alsaleh, 2024; Xiaojuan, 2023). The identification of Economics and Technology as moderators is a pivotal finding. Unlike family, these factors do not inherently create culture; instead, they act as ‘volume controllers’ that dictate the intensity of cultural transmission. Economic capital, for instance, determines the ‘gatekeeping’ of access to educational and political resources. Similarly, economic factors also serve as moderators, as the distribution of economic capital determines the extent to which individuals or groups can access education, media, and technology. Those with greater economic resources have a greater chance of maintaining or expanding their cultural influence ( Bourdieu, 1984).

Political and power factors show a pattern of dual relationships: direct and mediated. In a direct relationship, politics shapes cultural reproduction through education policies, language regulations, and national cultural policies. However, politics also serves as a mediator, channeling dominant ideologies and values into public consciousness. Gramsci (1971) emphasized that political hegemony operates through a combination of coercive domination and moral-intellectual leadership, so that the state and the political elite serve as the main link between the power structure and citizens’ cultural consciousness.

Finally, social and community environmental factors have a direct relationship with cultural reproduction. Everyday interactions within the community create a space for the formation of a collective identity, where norms, practices, and symbols are inherited and negotiated ( Anderson, 1991). These relationships are direct because communities provide a real social arena for cultural practice, without the need for formal institutional intermediaries. For instance, indigenous communities maintain vibrant cultural practices, such as ceremonies and intergenerational knowledge transfer, which reinforce community identity and values. These practices act as mechanisms for cultural transmission and social cohesion, emphasizing the role of community as a direct arena for cultural reproduction without institutional intermediaries (Torri & Herrmann, 2011; Tyagi & Jha, 2025).

The distribution of relationships shown in the heatmap reveals an interesting pattern: structural factors such as education, politics, and economics tend to play a role through mediation or moderation mechanisms, while socio-cultural factors such as family, religion, and community tend to be directly related to cultural reproduction. Media and technology occupy a transitional position, serving as both mediators and moderators. This indicates that cultural reproduction occurs through a combination of direct and indirect mechanisms, in which macro institutions play a greater role as liaisons or regulators, while microagents facilitate everyday transmission.

### Cultural reproduction

Cultural reproduction is the process by which social values, norms, symbols, and practices are passed down from one generation to the next. This happens through primary agents such as family, religion, and community, as well as through intermediary institutions and mechanisms such as education, media, politics, economics, and technology. This process not only preserves the current social structure but also facilitates the transformation and negotiation of meaning in response to an evolving social context. There are three types of relationships between factors: direct, mediated, and moderated. Together, these form a complex system for keeping culture alive. The interaction pattern of these factors is illustrated in Figure 4.

**Figure 4.**
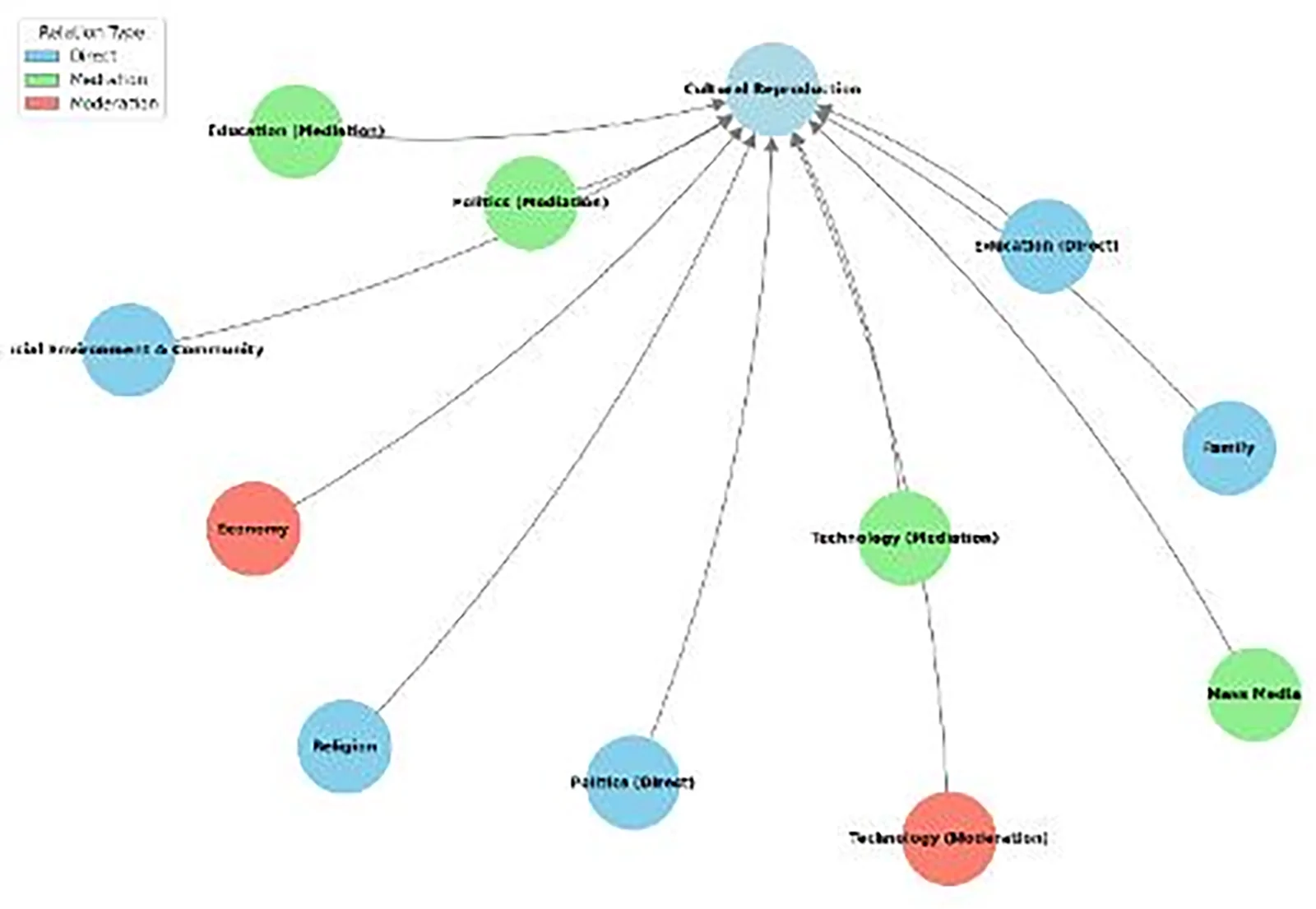
Cultural reproduction model based on ( Jiao et al., 2016) https://zenodo.org/records/18059311.

Cultural reproduction is a complex process that uses a range of mechanisms to maintain values, norms, and identities from one generation to the next. Conceptual diagrams derived from theoretical analysis indicate that social, political, economic, technological, and cultural factors operate not uniformly, but through distinct relationships: direct, mediation, and moderation. The differences in these kinds of relationships show that cultural reproduction occurs through interactions at different levels, where microagents, macro institutions, and contextual infrastructures all affect one another. To understand how culture survives and changes in modern society, we need to understand these different kinds of relationships better.

Family, religion, social community, and certain educational and political roles are directly associated with cultural reproduction. The family, as the principal agent of socialization, transmits habitus, language, and normative values from early childhood ( Bourdieu, 1977). Conversely, religion preserves the continuity of collective moral identity through symbols, rituals, and traditions grounded in quotidian existence ( Geertz, 1973). Social communities create a space for people to interact with each other and work out their shared cultural identities ( Anderson, 1991). Education and politics, in their direct relationship, dictate cultural curricula and policies that shape the propagation of prevailing values ( Bourdieu & Passeron, 1990; Gramsci, 1971). So, direct relationships show that both primary agents and macro institutions are crucial to ensuring that culture endures.

Education, mass media, politics, and digital technology are among the factors that can help with mediation. Education serves a dual role: it operates directly and also mediates between the family’s cultural capital and broader social opportunities. Schools not only replicate the classroom structure but also facilitate individual access to social resources ( Bourdieu, 1986). Mass media mediates symbolic representations that shape people’s perceptions of reality, providing material for the construction of identity and behavior ( Kellner, 1995). Politics, through ideology and policy, functions as a conduit that transmits prevailing values into societal consciousness ( Kaufusi, 2024). On the other hand, digital technology speeds up the flow of cultural exchange across borders, making virtual spaces a new place for meaning to be reproduced ( Castells, 2000). This mediation mechanism illustrates that cultural reproduction frequently occurs via intermediary channels that filter, modify, and disseminate specific values.

The moderation factor also plays a big role in controlling how strongly other factors affect things. The main factor controlling things is economics, because the distribution of economic capital determines how people or groups can access education, media, and politics. People who have more money are more likely to keep or grow their cultural influence, while people who don’t have much money are often pushed to the side ( Bourdieu, 1986). Digital technology also acts as a moderator, making other factors stronger or weaker depending on how it is used. Digital platforms, for instance, can expand access to online education or alter the dynamics of mass media information distribution, simultaneously creating opportunities for counterculture ( Kudelić et al., 2025). Moderation, then, is a contextual variable that affects the size and scope of cultural reproduction.

From the overall analysis, it is evident that cultural reproduction is neither linear nor static; rather, it arises from intricate interactions among various types of relationships. Directly related factors are the main sources of cultural value, and mediating factors ensure that those values are disseminated and change over time in society. Moderation, conversely, governs the intensity of influence, allowing cultural reproduction outcomes to differ across social contexts. This pattern aligns with Bourdieu’s (1986) theory of hegemony, which highlights the significance of ideological dominance in perpetuating cultural power. The role of technology and media as mediators and moderators aligns with Gramsci’s (1971) theory of hegemony, which highlights the significance of ideological dominance in perpetuating cultural power. The role of technology and media as mediators and moderators aligns with Castells’ (2000) examination of a networked society, wherein the circulation of digital information constitutes the primary foundation of social reproduction.

This conceptual diagram delineates the factors of cultural reproduction and underscores the need to differentiate the types of relationships that operate within the process. The interplay of direct relations, mediation, and moderation demonstrates that cultural reproduction is dialectical, emerging from the interaction among objective structures, subjective agents, and technological and economic contexts. These results indicate the need for a comprehensive analytical framework that views cultural reproduction as a multidimensional and evolving phenomenon. Only through this perspective can the examination of cultural reproduction yield a more comprehensive understanding of how societies sustain and evolve their cultural identities amid global transformation. The proposed model illustrates that cultural reproduction is a dialectical system in which micro-agents provide the content of culture, macro-institutions provide legitimacy, and moderating variables define the reach of that culture in a modern, networked society.

## Discussion

### 1. Interpretation of main findings and their relation to research objectives

The systematic review of 143 arrticles demonstrates that cultural reproduction is a complex process operating through multi-level interactions between micro agents and macro structures. These findings confirm that cultural reproduction functions as more than a mere tool for maintaining exicting social hierarchies. Instead, it facilitates the adaptation and transformation of values according to dynamic social contexts. The distribution of factors indicates that education, politics, and family are the primary agents sustaining cultural continuity. ducation performs a dual role by acting as an instrument for legitimizing dominant values through curriculum and simultaneously serving as a mediator between family cultural capital and social opportunities. Overall, these findings confirm that cultural reproduction not only serves to maintain existing social structures but also allows adaptation and transformation of values according to dynamic social contexts. Thus, these results successfully answer the research objectives of analyzing the factors that influence cultural reproduction, explaining the main concepts, and formulating a conceptual model that links them to socio-cultural resilience.

The distribution of factors shows that education, politics, and family are the dominant agents in maintaining cultural sustainability. Education plays a dual role: first, as an instrument of legitimization of dominant values through curriculum and pedagogy (direct reproduction); Second, as a mediator between family cultural capital and social opportunities (indirect reproduction). Politics and economics work to regulate the distribution of capital that determines access to cultural resources, while media and digital technology are becoming new links in the transmission of values across geographical and generational boundaries. Thus, the cultural reproduction model formed is integrative-adaptive, combining direct relationships, mediation, and moderation among the eight main factors. These findings strengthen the position of the research as a theoretical and empirical synthesis that bridges various disciplinary domains in understanding socio-cultural resilience.

### 2. Comparison with previous literature

a. Alignment with Previous Research
  The findings are consistent with the classical framework of Bourdieu (1977, 1986) which emphasizes the role of education and family in the inheritance of cultural capital. Schools remain a dominant arena that reproduces the habitus of the middle class through the symbolic legitimacy of the curriculum. Harmony is also seen with Gramsci’s (1971) view of hegemony, in which ideology and politics function to maintain class dominance through social consent, not coercion. In the contemporary context, these results support the findings of Janssen et al. (2024) suggesting that cultural reproduction now occurs within digital spaces where algorithms act as new symbolic mediators.
  The study of Ghahramani et al. (2020) and Lv et al. (2025) also strengthens the evidence that cultural heritage and community participation contribute directly to social resilience, especially when integrated with participatory governance and sustainable tourism. Thus, the results of this systematic review confirm the causal relationship between cultural reproduction and socio-cultural resilience, as indicated in the cross-contextual empirical literature.
b. Critical Departures and Contradictions with Literature
  Although in line with classical theory, the results of this study also show an important deviation from Bourdieu’s deterministic approach. In many contemporary studies, it has been found that cultural reproduction not only maintains inequality, but also opens up space for social mobility, as argued by DiMaggio (1982) and Blaskó (2003). While this study acknowledges classical foundations, the results show a significant departure from the deterministic approach often associated with Bourdieu. Our synthesis aligns more closely with Ann Swidler’s (1986) perspective on the Cultural Tool Kit, suggesting that individuals are not passive recipients of culture but actively select cultural elements to negotiate their social positions. This implies that cultural capital has evolved into a transformative instrument rather than a purely reproductive one.
  Regarding technology, the study finds that digital platforms function as more than neutral mediators. This phenomenon can be critically analyzed through the lens of Couldry and Mejias (2019) regarding data colonialism, where algorithms act as invisible gatekeepers that prioritize globalized narratives. This forces local or indigenous cultures to struggle for symbolic visibility against automated structural biases. While (Yosso, 2005) conceptualizes technology as a neutral structural mediator, the results of this study indicate that technology functions as a moderator that regulates the intensity and reach of cultural reproduction. Unlike a mere conduit, technology operates as a volume controller where high digital access may amplify the transmission of traditional values, yet the digital divide acts as a structural barrier that weakens the reproductive capacity of marginalized groups. This interaction effectively expands the framework of network theory by adding a dimension of social amplification to the process of cultural reproduction.
c. Thematic Synthesis and New Contributions
  This study introduces a systemic approach to socio-cultural resilience by positioning cultural reproduction as a dynamic instrument of adaptation rather than a static process of preservation. A critical addition to the literature is the integration of Yosso (2005) Community Cultural Wealth, which fundamentally challenges the traditional Bourdieusian view that often overlooks the organic strengths of marginalized groups. While education remains the most dominant factor at 17.6%, this dominance can lead to symbolic exclusion if formal curricula continue to devalue the inherent cultural capital specifically the linguistic, navigational, and resistant capital of marginalized or indigenous communities. In the context of indigenous groups, the relationship between the community environment and formal education must be reciprocal. Schools should not merely act as sites of dominant value transmission but must recognize and integrate local wisdom as a legitimate form of knowledge to prevent cultural erosion, ensuring that the habitus of the community is treated as a unique asset that enhances social-community resilience.
  Furthermore, the relationship between these factors is increasingly governed by the urgent need for socio-ecological sustainability. By linking cultural reproduction to environmental survival, as suggested by Adger et al. (2013), this study positions cultural transmission as a vital survival strategy for communities facing global ecological crises. The interaction between family, religion, and the social environment involves the active preservation of traditional ecological knowledge, where technology and media must function as amplifiers rather than neutral conduits to ensure the “volume” of indigenous sustainability practices contributes to global resilience strategies. Consequently, the strength of cultural reproduction depends on a synergy where the preservation of indigenous habitus becomes a prerequisite for navigating external disruptions like globalization and climate change. This systemic interaction asserts that cultural continuity is the foundation upon which communities build their capacity to survive and thrive amidst ongoing environmental transformations.

### 3. Thematic synthesis of each research question

a. Main Concepts of Cultural Reproduction
  The literature synthesis shows that cultural reproduction is a mechanism of inheritance of values and norms that works through symbolic structures, social institutions, and everyday interactions. While Bourdieu’s (1986) foundational concepts of habitus and symbolic violence remain relevant, this study finds that they are insuffient to explain the transformative elasticity of culture in the 21 ^st^ century. However, contemporary contexts extend it through the digital, participatory, and intersectionality dimensions of identity ( Crenshaw, 1991). Cultural reproduction now takes place not only through education and family, but also through digital platforms that determine symbolic visibility and legitimacy. Therefore, the reproduction of modern culture can be understood as a dialectical process between conservation and innovation in the value system of society.
b. Factors Affecting Cultural Reproduction
  The systematic analysis identified eight main factors: family, education, politics, economy, religion, mass media, technology, and the social-community environment. Education occupies the most dominant position (17.6%), followed by politics (15.3%) and family (14.1%). Each factor has a different type of relationship: direct, mediated, or moderate. Micro factors such as family and religion work directly, while macro factors such as politics, media, and economics function through intermediaries or arrangements of the intensity of influence. This synthesis suggests that cultural reproduction cannot be understood linearly but as a social ecological system that influences each other.
c. Relationships between Factors
  The findings suggest that the interaction between factors forms a dynamic network. Education is the main node that connects the family’s cultural capital with economic and political opportunities. Media and technology mediate the spread of cultural symbols across regions, while economics and technology serve as moderators that determine the scale and extent of the influence of other factors. This pattern reinforces Bourdieu’s (1986) theory of compound capital while complementing Castells’ (2000) network theory by adding a normative layer. Cultural reproduction is thus a relational process that moves through interdependence between social factors.
d. Cultural Reproduction Model as a Form of Socio-Cultural Resilience
  The model developed illustrates that cultural reproduction contributes directly to socio-cultural resilience through three mechanisms: (1) preservation of core community values, (2) adaptation to social and technological change, and (3) symbolic legitimacy of collective identity. Socio-cultural resilience is formed when cultural values and practices are able to maintain social cohesion while adapting to external dynamics such as globalization or environmental crises. This model asserts that resilience does not depend on conservation alone, but on the ability of communities to integrate tradition with innovation.

### 4. Theoretical, practical, and policy implications

a. Theoretical Implications
  These findings advance Bourdieu’s framework by demonstrating that cultural capital possesses a transformative elasticity, moving beyond static reproduction. This necessitates a shift toward a ‘Cultural Ecology’ theory, which views social resilience as the emergent result of systemic interactions between inherited habitus, political structures, and digital-mediated agency. Consequently, the definition of cultural capital must be expanded to include ‘Digital Capital’ as a prerequisite for symbolic visibility in the 21st century.
b. Practical Implications
  Practically, the results of this study provide a basis for designing adaptive cultural preservation programs. Education must be directed at the transmission of values across generations through the integration of a curriculum based on local wisdom and digital literacy. Governments and cultural institutions can use this model to strengthen community participation in the preservation of intangible cultural heritage (ICH) as advocated by McDermott (2024) and Sun et al. (2024). At the community level, this model can be applied to increase cultural awareness through interactive activities based on digital media, which combine tradition with technological innovation.
c. Policy Implications
  For policymakers, these findings underscore the importance of participatory governance and cross-sectoral collaboration. Cultural preservation policies cannot be top-down but need to be designed as an adaptive cultural policy ecosystem that connects education, the creative economy, and digital technologies. In addition, socio-cultural resilience indicators need to be developed by considering the dimensions of community participation, equity of access to cultural resources, and environmental sustainability. Thus, the results of this study support UNESCO’s policy direction in strengthening the role of cultural heritage as a source of global resilience.

### 5. Unexpected findings and explanations

One unexpected result is the emergence of the ambivalent role of digital technology in the process of cultural reproduction. Although it is expected to be a means of democratizing culture, the results of the synthesis suggest that technology can also reinforce inequality through algorithms that focus on dominant cultures. This phenomenon explains how social media can reproduce symbolic hegemony in a new format. This phenomenon illuminates the rise of ‘Algorithmic Hegemony’, where digital platforms, under the guise of democratization, often prioritize dominant global narratives, effectively reproducing symbolic violence through automated filtering. However, the ‘ambivalence’ of this digital space also provides a subaltern arena for minority cultures to build global solidarity, suggesting that digital reproduction is a site of constant ideological contestation. But on the other hand, the digital space also opens opportunities for minority cultures to express their identities and build a global solidarity network. This ambivalence confirms that digitalization is not just an instrument, but a new ideological arena that needs to be critically examined.

In addition, another interesting finding is the role of local communities that has tended to be overlooked in previous literature. Community turns out to play a strategic function in maintaining a balance between value conservation and social adaptation. The practice of cooperation, rituals, and traditional arts are a source of moral strength that sustains social resilience amid modernization pressure. This shows that resilience is not only built through macro policies, but also through micro-social networks that live in people’s daily lives.

### 6. Methodological strengths and limitations

The Systematic Literature Review (SLR) approach is a major strength of this research because it allows for cross-disciplinary integration in a transparent and replicative manner. The use of the PRISMA protocol and MMAT tool ensures the validity of the selection and the reliability of the interpretation of the results. Thematic analysis using NVivo deepens the understanding of the relationships between factors and enables the development of evidence-based conceptual models. However, there are some limitations. First, the dominance of English-language articles has the potential to exclude local non-English literature that is relevant to Asian or African contexts. Second, the analysis only covers the 2015–2025 range, so it does not fully capture the historical dynamics of long-term cultural reproduction. Third, although SLR provides a strong theoretical synthesis, this study does not include direct empirical validation through field observations or in-depth interviews. Therefore, the results are conceptual and need to be tested in a specific empirical context.

### 7. Knowledge gap and direction of advanced research

This study reveals several gaps in knowledge that open up opportunities for further study. First, there is still limited research on the reproduction of digital culture in developing countries, even though online space is now the main arena for cultural interaction. Second, there is a need for cross-generational empirical studies that observe the transmission of cultural values from families to digital communities, including the dynamics of resistance and identity hybridization. Third, a significant gap exists in the intersection of cultural reproduction and long-term socio-environmental sustainability. Specifically, there is an urgent need to investigate how traditional knowledge and local wisdom are systematically reproduced to maintain community survival amidst global environmental crises. Future research should examine whether the inheritance of these specific cultural values can mitigate the negative impacts of external disruptions on social cohesion.

To fill these gaps, future research can combine mixed-methods with a community-based participatory approach. This approach will allow for empirical validation of the developed model while enriching the theory with contextual data. Interdisciplinary studies between cultural sociology, communication technology, and policy studies also need to be expanded to understand new dynamics in global cultural reproduction.

This discussion emphasized that cultural reproduction is the main foundation of socio-cultural resilience. Through the complex interaction between micro-agents and macro structures, cultural values and identities are not only maintained but also adapted in the face of global change. This research expands on classical theory by adding digital, participatory, and social ecology dimensions that make cultural reproduction a mechanism of collective resilience. With an integrative model that combines direct relationships, mediation, and moderation, the results of this study make new contributions to the theory, practice, and policy of cultural preservation. Through this synthesis, cultural reproduction is understood not only as a process of preserving the past, but as a future adaptive strategy for society to survive and develop in the ongoing flow of globalization and technological transformation.

This section should be completed as per the PRISMA checklist, item 23. (For scoping reviews, see PRISMA-Scr, items 19-21).

## Conclusion

This study, synthesized from 143 peer-reviewed articles, establishes that cultural reproduction is a multidimensional, nonlinear process driven by the interplay between micro-agents and macro-structures. The findings redefine cultural reproduction not as a static preservation mechanism, but as a dynamic adaptive strategy that fosters socio-cultural resilience. By integrating eight pivotal factors through direct, mediated, and moderated pathways, this research introduces a “cultural reproductive ecosystem” that evolves in tandem with social and technological shifts. Furthermore, this study advances Bourdieusian theory by incorporating digital, participatory, and socio-ecological dimensions, positioning cultural capital as a transformative tool for social sustainability. Ultimately, this theoretical synthesis bridges the gap between classical reproduction theories and modern resilience frameworks, asserting that culture is the foundational pillar for collective identity and long-term societal survival.

### Suggestion

Despite providing a robust conceptual framework, this study acknowledges several methodological constraints, including the predominance of English-language literature, the specific timeframe (2015–2025), and the lack of direct empirical validation for the proposed model. To address these limitations, the following trajectories for future research and policy are suggested: Future studies should employ mixed-methods approaches, including longitudinal field observations and participatory interviews. Research is needed to decode the transmission of cultural values in the digital era, with a specific focus on under-represented non-Western and indigenous contexts to counter-balance the current Western-centric discourse. Subsequent research should investigate the synergy between cultural reproduction and environmental resilience, exploring how traditional knowledge systems, such as those found in indigenous communities, adapt to climate change and global crises. For policymakers, these findings highlight the necessity of a participatory cultural policy ecosystem. This ecosystem must integrate inclusive education, digital literacy, the creative economy, and the safeguarding of intangible heritage to ensure cultural sustainability.

## Data Availability

No data associated with this article. Repository name: Dataset Article SLR: Models Of Cultural Reproduction As Drivers Of Socio-Cultural Resilience: Insights From A Systematic Review https://zenodo.org/records/18058118 and https://zenodo.org/records/18059311 ( Al Faqi, et al., 2025). This project contains the following extended data:
1. Dataset Selection Process (Collection of article data used for literature review) 2. PRISMA 2020 Checklist 3. Dataset Graphic Figure 2 4.
Figures 1, 2, 3 and 4 Repository name: PRISMA checklist for Models Of Cultural Reproduction As Drivers Of Socio-Cultural Resilience: Insights From A Systematic Review https://zenodo.org/records/18058118 ( Al Faqi, et al., 2025). Data are available under the terms of the Creative Commons Attribution 4.0 International license (CC-BY 4.0).
