# Supplementary material for: Models of Cultural Reproduction as Drivers of Socio-cultural Resilience: Insights from a Systematic Review
Source: F1000Res. 2026 May 25;15:72. Originally published 2026 Jan 16. [Version 2] doi: 10.12688/f1000research.174513.2 (PMC13624493; doi:10.12688/f1000research.174513.2)
Supplement: Peer review history

# Reviewer report: Reviewer response for version 2

**DOI:** 10.5256/f1000research.197743.r524350

**Published:** 2026-09-29

**Authors:** Apostolos Karaoulas

**Affiliations:** 1 University of Ioannina, Ioannina, Greece

This Systematic Literature Review (SLR) explores the mechanisms of cultural reproduction as an adaptive strategy to the challenges of globalization, digitization, and climate change that societies experience today. A total of 143 articles published between 2015 and 2025 were analyzed to identify the key factors that contribute to the development of socio-culturally resilient communities. The study revealed an eight-determinant framework consisting of education, politics, family, economy, media, technology, religion, and community.

 The second version of the manuscript is polished and improved, providing readers with a clearer and more complete picture. It incorporates critical theoretical elements that reduce rigid determinism by emphasizing the role of technology. The research questions and the objective of the study are defined clearly and concisely, maintaining a sharp focus on societies’ ability to reproduce their cultural resources.

 The study’s reliability and validity are well supported by the authors’ thorough description of the search and selection strategy, as well as the quality assessment process using the MMAT tool. Both PRISMA and PICO protocols were applied to screen and include the 143 articles in the final analysis, providing all the necessary details for full replication. As a qualitative or mixed-methods systematic review, using descriptive statistics to present the frequency distribution of each determinant is fully justified.

 The findings section derives directly from the analyzed body of work. Specifically, the eight-determinant framework was identified through the thematic analysis of the 143 empirical articles. Regarding Question 5, this study qualifies as a classic, static systematic review with a defined timeframe (2015-2025), rather than a «Living Systematic Review». As a result, the response is marked as Not Applicable.

 Regarding the optional suggestions and theoretical reflections that the authors might include, either in the present manuscript or as directions for future research, it would be particularly valuable to highlight the relationship between globalization and cultural reproduction. Although the article correctly identifies globalization, algorithmic hegemony, and data colonialism as sweeping pressures, the proposed model views cultural reproduction strictly as an inherent mechanism of adaptive resilience. However, there is a theoretical blind spot here: it is almost taken for granted that cultural reproduction can always act as an adequate counterweight, underestimating how destructive globalization can be in regions with a history of violent colonialism where local social structures were irreparably disrupted.

 In this light, examining historical depth in future work is recommended, as culture is not merely a tool for present adaptation, but part of an organic historical continuity. This continuity develops differently in societies with an uninterrupted, indigenous cultural trajectory, such as in Europe or East Asia (e.g., China and Japan), compared to regions that suffered violent colonial enforcement, such as in Africa or Latin America, where intergenerational transmission was broken or reshaped by colonial trauma. Finally, providing a clearer typological clarification of intangible cultural heritage in future research would be useful, highlighting which specific forms, such as traditional ecological knowledge versus language preservation, surfaced most frequently during the analysis of empirical studies.

Are the rationale for, and objectives of, the Systematic Review clearly stated?

Yes

Is the statistical analysis and its interpretation appropriate?

Yes

If this is a Living Systematic Review, is the ‘living’ method appropriate and is the search schedule clearly defined and justified? (‘Living Systematic Review’ or a variation of this term should be included in the title.)

Not applicable

Are sufficient details of the methods and analysis provided to allow replication by others?

Yes

Are the conclusions drawn adequately supported by the results presented in the review?

Yes

Reviewer Expertise:

My research interests and academic specialization cover the History and Sociology of Education, the study of Educational Institutions in general (from primary to higher education), the interplay between Education and Culture, mechanisms of Symbolic Power, as well as Conceptual History / the History of Concepts in Education (Begriffsgeschichte des Bildungswesens)

I confirm that I have read this submission and believe that I have an appropriate level of expertise to confirm that it is of an acceptable scientific standard.

# Reviewer report: Reviewer response for version 2

**DOI:** 10.5256/f1000research.197743.r489961

**Published:** 2026-06-22

**Authors:** Kusnul Khotimah, Katon Galih Setyawan

**Affiliations:** 1 Universitas Negeri Surabaya, Surabaya, Indonesia; 2 Social Studies, Universitas Negeri Surabaya, Surabaya, East Java, Indonesia

This article is highly accurate, well-structured, and fully deserving of indexing in a reputable academic journal. The study demonstrates a strong methodological foundation and presents findings that are both credible and valuable for future research. The analysis is exceptionally thorough, supported by relevant and up-to-date theoretical frameworks that strengthen the interpretation of the results. Furthermore, the application of the PICO and PRISMA approaches have been conducted appropriately and in accordance with established international standards for systematic review research. The research procedures are clearly described, ensuring transparency, reliability, and reproducibility. As a result, other researchers can confidently replicate the study in similar or different contexts. Overall, the article meets high scholarly standards and presents no significant methodological or conceptual weaknesses that would raise concerns regarding its quality or validity.

Reviewer Expertise:

Social Studies Learning; Values ​​Education in Social Studies Learning; Character building

We confirm that we have read this submission and believe that we have an appropriate level of expertise to confirm that it is of an acceptable scientific standard.

# Response

**Published:** 2026-06-29

**Authors:** Mifdal Zusron Alfaqi

**Affiliations:** Ilmu Ketahanan Nasional, Universitas Brawijaya, Malang, East Java, Indonesia

Thank you very much for your positive review and valuable comments.

 We sincerely appreciate your time and thoughtful evaluation of our manuscript. Your encouraging feedback is greatly appreciated and motivates us in our future research.
